# A machine learning-based predictive model for postoperative pulmonary complications in lung cancer and its SHAP interpretation

**DOI:** 10.3389/fonc.2026.1749808

**Published:** 2026-03-13

**Authors:** Yun Sha, Zejing Huangfu, Xinyu Gu, Beining Tang, Zhenchao Lv, Yanming Li, Ji Yang, Jinyuan Yang, Shihao Shao, Zhonghui Wang

**Affiliations:** 1 Department of Anesthesiology, The Third Affiliated Hospital of Kunming Medical University, Yunnan Cancer Hospital, Peking University Cancer Hospital Yunnan, Kunming, China; 2 Department of Anesthesiology, Simao District People’s Hospital of Pu’er City, Pu’er, China

**Keywords:** lung cancersurgery, machine learning models, postoperative pulmonary complications (PPCs), risk prediction, SHAP interpretation

## Abstract

**Background:**

Postoperative pulmonary complications (PPCs) significantly impair patient recovery and adversely affect the long-term prognosis following lung cancer surgery. Despite ongoing advancements in surgical techniques and perioperative care, the incidence of PPCs remains elevated, underscoring the pressing clinical necessity for dependable preoperative risk assessment tools.

**Methods:**

This study employed a retrospective design, encompassing 1, 223 patients who underwent lung cancer surgery, from whom perioperative clinical data were collected. Following data cleansing and feature selection, the dataset was stratified and randomly divided into training (70%) and testing (30%) sets. Model development and hyperparameter tuning were executed using stratified 10-fold cross-validation (CV) within the training set; all preprocessing and feature selection procedures were confined to the training folds to prevent information leakage. The discriminative and calibration performance of various machine learning algorithms were assessed, and clinical net benefits were appraised using decision curve analysis (DCA). Additionally, Shapley Additive Explanations (SHAP) were employed to elucidate the contributions of specific features to the risk of developing PPCs.

**Results:**

Among the evaluated models, the k-nearest neighbors (KNN) algorithm demonstrated superior performance, evidenced by a high area under the receiver operating characteristic curve (AUROC) and favorable clinical utility in the DCA. SHAP analysis revealed that factors such as perioperative inflammatory burden, diabetes, hypertension, and smoking history are pivotal in influencing the risk of PPCs.

**Conclusion:**

The developed machine learning-based predictive model, augmented with SHAP interpretations, effectively identifies patients at high risk for PPCs prior to surgery. This model provides a robust scientific foundation for tailored perioperative care and interventions, offering substantial potential for clinical application.

## 1 Introduction

Lung cancer constitutes one of the malignant neoplasms with the highest incidence and mortality rates worldwide (1). Surgical resection remains a pivotal strategy for curing or significantly prolonging survival in certain patients with non-small cell lung cancer (2). Nevertheless, the prevalence of postoperative pulmonary complications (PPCs) in clinical settings is considerable, typically ranging from 7.4% to 48%, with an average incidence of approximately 18.4% (3, 4). Common PPCs encompass atelectasis, pneumonia, respiratory failure, and aspiration pneumonia (5). These complications, which are heterogeneous in nature, markedly impair patient prognosis, as evidenced by prolonged hospital stays, escalated healthcare costs, increased readmission rates, and heightened mortality risks both in the short and long term (6, 7). The preoperative identification of high-risk individuals to facilitate targeted perioperative optimization and interventions constitutes a fundamental challenge in enhancing surgical outcomes and the efficiency of resource allocation (8, 9).

The existing research on PPCs in lung cancer is diverse. Traditional statistical models and empirical scoring systems, such as nomograms or logistic regression (LR) models, which are based on perioperative baseline information and laboratory indicators, have demonstrated moderate discriminative power in several single-center studies. These models show some predictive ability for common complications such as pneumonia and atelectasis (10, 11). However, many studies suffer from limited sample sizes and lack sufficient external validation. They also have incomplete reporting of calibration and decision-related statistical metrics, which limits their generalizability and clinical applicability (12, 13). In recent years, machine learning has exhibited superior discriminative ability compared to traditional methods in medical prediction, capable of uncovering nonlinear relationships and interaction effects within high-dimensional feature spaces (14). Nevertheless, the insufficient interpretability of these models often poses a barrier to their clinical implementation (15). Shapley Additive Explanations (SHAP), as a game theory-based interpretative framework that is consistent both globally and locally, can quantify the marginal contribution of individual features to both individual predictions and overall model outputs. This provides a powerful tool for enhancing model transparency and clinical interpretability (16, 17). Currently, research focused on the prediction of PPCs predominantly utilizes traditional statistical models, accompanied by a limited array of disparate machine learning initiatives. Notably, this field faces challenges including a dearth of external validation, opacity in the modeling processes, and an absence of systematic, multi-algorithm comparative evaluations. While inflammatory and immune-related indicators are promising for predicting PPCs, their optimal thresholds and applicability exhibit considerable variability across diverse populations, making it challenging to achieve a unified and comprehensive risk assessment (18).

This study aims to conduct a systematic benchmark comparison of various machine learning models using a single-center retrospective cohort. It evaluates the discriminative and calibration performance through stratified cross-validation (CV) and independent test sets, employing consistent data and processes. By integrating SHAP, the study furnishes both global and individual-level interpretability, identifying key perioperative risk factors. Additionally, decision curve analysis (DCA) is employed to assess the clinical net benefits at different thresholds, aiming to select PPCs prediction tools that balance performance and feasibility. This approach provides a quantitative basis for risk stratification and individualized interventions in preoperative anesthetic assessments.

## 2 Methods

### 2.1 Data collection

The protocol for this retrospective cohort study has been approved by the Clinical Research Ethics Committee of the Third Affiliated Hospital of Kunming Medical University (KYLX2025-162). Due to the retrospective design of the study and the anonymization of the data, the requirement for informed consent was waived. From January 2024 to January 2025, a retrospective collection of data was conducted on surgical patients from the Department of Thoracic Surgery at the Third Affiliated Hospital of Kunming Medical University, totaling 1, 223 patients. After the exclusion of 119 patients who underwent non-lung cancer surgeries, 4 minors, and 3 patients with severe data deficiencies, data from a total of 1, 097 patients were included in the analysis. The collected data encompass patient characteristics, laboratory indicators, tumor size, duration of surgery, and surgical site information (**Table 1**).

**Table 1:** Baseline characteristics of clinical data.

Variable	Total (N = 1097)	PPCs=No (N = 816)	PPCs=Yes (N = 281)	P-value
Gender, n(%)				
Female	642 (58.5)	511 (62.6)	131 (46.6)	<0.001
Male	455 (41.5)	305 (37.4)	150 (53.4)	
PSH, n(%)				
No	922 (84.0)	672 (82.4)	250 (89.0)	0.012
Yes	175 (16.0)	144 (17.6)	31 (11.0)	
Hypertension, n(%)				
No	912 (83.1)	705 (86.4)	207 (73.7)	<0.001
Yes	185 (16.9)	111 (13.6)	74 (26.3)	
Diabetes, n(%)				
No	945 (86.1)	730 (89.5)	215 (76.5)	<0.001
Yes	152 (13.9)	86 (10.5)	66 (23.5)	
CHD, n(%)				
No	1071 (97.6)	798 (97.8)	273 (97.2)	0.702
Yes	26 (2.4)	18 (2.2)	8 (2.8)	
Smoke, n(%)				
No	805 (73.4)	654 (80.1)	151 (53.7)	<0.001
Yes	292 (26.6)	162 (19.9)	130 (46.3)	
Surgical site, n(%)				
Right	623 (56.8)	460 (56.4)	163 (58.0)	0.684
Left	474 (43.2)	356 (43.6)	118 (42.0)	
LND, n(%)				
No	566 (51.6)	464 (56.9)	102 (36.3)	<0.001
Yes	531 (48.4)	352 (43.1)	179 (63.7)	
ASA PS, n(%)				
I	4 (0.4)	2 (0.2)	2 (0.7)	<0.001
II	978 (89.2)	747 (91.5)	231 (82.2)	
III	113 (10.3)	67 (8.2)	46 (16.4)	
IV	2 (0.2)	0 (0.0)	2 (0.7)	
Age, mean (SD)	55.61 (10.78)	54.38 (10.73)	59.17 (10.11)	<0.001
BMI, mean (SD)	23.51 (3.19)	23.46 (3.13)	23.65 (3.35)	0.403
Operation time, mean (SD)	83.32 (35.92)	77.37 (31.36)	100.58 (42.25)	<0.001
Anesthesia time, mean (SD)	100.92 (36.91)	95.37 (32.82)	117.02 (42.99)	<0.001
WBC, mean (SD)	6.20 (1.89)	6.26 (1.94)	6.02 (1.73)	0.061
SIRI, mean (SD)	1.02 (1.19)	0.80 (0.51)	1.69 (2.04)	<0.001
PLT, mean (SD)	241.04 (68.64)	240.86 (68.23)	241.57 (69.92)	0.881
HB, mean (SD)	148.17 (16.30)	148.79 (15.94)	146.36 (17.21)	0.031
ALB, mean (SD)	45.18 (3.29)	45.27 (3.07)	44.92 (3.84)	0.122
FEV1%, mean (SD)	99.25 (18.20)	99.19 (18.13)	99.43 (18.45)	0.847
DLCO%, mean (SD)	106.97 (22.98)	107.11 (22.73)	106.58 (23.74)	0.742

The inclusion criteria were established as follows (1): patients diagnosed with lung cancer, confirmed through pathological examination; (2) patients aged 18 years or older; (3) patients possessing comprehensive clinical and pathological data. The exclusion criteria encompassed: (1) patients suffering from significant comorbidities that could severely distort the outcomes; (2) patients with severe immunodeficiency diseases; (3) patients who encountered major technical complications during surgery, which precluded completion of the procedure as planned; (4) patients with intellectual disabilities or other severe mental health conditions. Furthermore, postoperative complications identified included atelectasis, pulmonary infections, chylothorax, persistent air leaks, persistent pleural effusion (defined as drainage time exceeding five days), pneumothorax, subcutaneous emphysema, and hemothorax.

### 2.2 Data preprocessing

A preliminary analysis was conducted on 20 clinical characteristics. Among these characteristics, the categorical variables included gender, history of surgery, hypertension, coronary artery disease, diabetes, smoking history, surgical site, status of intraoperative lymph node dissection (LND), and the American Society of Anesthesiologists physical status classification (ASA); whereas the remaining variables were continuous and included age, body mass index (BMI), duration of surgery, duration of anesthesia, white blood cell count, systemic inflammatory response syndrome score, platelet count, hemoglobin (HB) level, albumin level, forced expiratory volume in one second (FEV1%), and diffusing capacity of the lungs for carbon monoxide (DLCO%). For variable-related information, see **Supplementary Table 1**. Specifically, SIRI was calculated strictly based on baseline results from the preoperative complete blood count with differential, using the absolute neutrophil, monocyte, and lymphocyte counts from the test performed within 7 days before surgery or the most recent preoperative test, according to the formula SIRI = Neutrophils × Monocytes/Lymphocytes.This study did not include intraoperative or immediate postoperative dynamic indicators.If a complete blood count was performed on the morning of surgery or prior to entering the operating room and it was the last preoperative test, the result was used as the baseline input to more accurately reflect the preoperative inflammatory status at the time point closest to surgery.

In this study, LND was included as a binary variable based on whether it was performed (1 = yes, 0 = no), and no further fine-grained information was recorded regarding the extent of dissection—such as systematic mediastinal lymph node dissection versus lymph node sampling, the number of stations, or the dissection field.The definitions of the relevant variables have been clearly specified in **Supplementary Table 1**. It should be emphasized that LND is an intraoperative procedural variable, and its dissection extent is primarily determined by the surgeon through an integrated assessment of clinical needs, intraoperative findings, disease burden, and operative complexity; therefore, confounding by indication may be present.Accordingly, LND was incorporated into the model to enhance the accuracy and practical utility of predicting perioperative PPCs, rather than to interpret it as a modifiable preoperative risk factor for patients.

In the analysis of categorical variables, all variables except for the ASA were coded as “1” to indicate the presence or occurrence of a condition and “0” to denote the absence or non-occurrence. The ASA was categorized distinctly as “1”, “2”, “3”, and “4”. Continuous variables were standardized using Z-scores. We chose missForest (random forest–based iterative imputation) because it is nonparametric, can capture nonlinear relationships and interactions, and is suitable for datasets containing both continuous and categorical clinical variables. The distribution of key variables showed no meaningful change before vs. after imputation (**Supplementary Figure 1**). Descriptive statistics were subsequently performed on the organized data (**Supplementary Figures 2, 3**), with all data processing executed using the R programming language (version 4.4.2).

### 2.3 Key feature selection

This study utilizes univariate and multivariate LR analyses to screen the features incorporated into the model, ultimately selecting those that demonstrate significant statistical relevance in either the univariate or multivariate contexts (**Table 2**).

**Table 2:** Results of univariate and multivariate regression analysis.

Variable	Group	Univariable OR (95%CI, P)	Multivariable OR (95%CI, P)
Gender	Female		
	Male	1.92 (1.46-2.52, p<.001)	0.94 (0.63-1.40, p=.755)
PSH	No		
	Yes	0.58 (0.38-0.88, p=.010)	0.53 (0.32-0.86, p=.010)
Hypertension	No		
	Yes	2.27 (1.63-3.17, p<.001)	2.12 (1.42-3.16, p<.001)
Diabetes	No		
	Yes	2.61 (1.83-3.72, p<.001)	2.73 (1.79-4.18, p<.001)
CHD	No		
	Yes	1.30 (0.56-3.02, p=.543)	
Smoke	No		
	Yes	3.48 (2.60-4.65, p<.001)	2.97 (1.97-4.48, p<.001)
Surgical.site	Right		
	Left	0.94 (0.71-1.23, p=.633)	
LND	No		
	Yes	2.31 (1.75-3.06, p<.001)	2.01 (1.43-2.82, p<.001)
ASA.PS	I		
	II	0.31 (0.04-2.21, p=.242)	
	III	0.69 (0.09-5.05, p=.712)	
	IV		
Age	Mean ± SD	1.05 (1.03-1.06, p<.001)	1.04 (1.02-1.06, p<.001)
BMI	Mean ± SD	1.02 (0.98-1.06, p=.403)	
Operation.time	Mean ± SD	1.02 (1.01-1.02, p<.001)	1.05 (1.03-1.08, p<.001)
Anesthesia.time	Mean ± SD	1.02 (1.01-1.02, p<.001)	0.96 (0.94-0.99, p=.001)
WBC	Mean ± SD	0.93 (0.86-1.00, p=.061)	
SIRI	Mean ± SD	2.08 (1.74-2.49, p<.001)	1.92 (1.59-2.31, p<.001)
PLT	Mean ± SD	1.00 (1.00-1.00, p=.881)	
HB	Mean ± SD	0.99 (0.98-1.00, p=.032)	0.99 (0.98-1.00, p=.231)
ALB	Mean ± SD	0.97 (0.93-1.01, p=.125)	
FEV1%	Mean ± SD	1.00 (0.99-1.01, p=.847)	
DLCO%	Mean ± SD	1.00 (0.99-1.00, p=.742)	

### 2.4 Machine learning model

This study employs a range of algorithms to construct predictive models, including LR, Decision Tree (DT), Random Forest (RF), KNN, Support Vector Classification (SVC), Neural Networks (NN), Extreme Gradient Boosting (XGBoost), and Light Gradient Boosting Machine (LightGBM), selected from a total of 12 machine learning algorithms. All machine learning analyses were conducted using R version 4.4.2. The research sample was randomly divided into a training set (n=768) and a test set (n=329), adhering to a 7:3 ratio. During the training phase, a 10-fold CV method was employed to evaluate model performance, while the test set underwent evaluation using metrics such as AUC, standard curves, and DCA curves.

The primary intended application of this study is to predict the risk of perioperative PPCs. In this context, false-negative results may delay the identification of high-risk patients, and their clinical cost is typically higher than that of false-positive results.Therefore, in addition to reporting model performance under the default threshold of 0.5, we adjusted the classification threshold without altering the model training process.Specifically, the predicted probabilities output by the KNN model on the test set were converted into class labels using cutoff = 0.5 (default) and cutoff = 0.3 (screening-oriented threshold), and performance metrics—including sensitivity, specificity, PPV, NPV, and overall accuracy—were compared to evaluate the model’s clinical applicability.

Given that the incidence of PPCs in this dataset was approximately 25.6%, class imbalance was present.We further conducted a SMOTE sensitivity analysis using the training set only.Specifically, categorical variables in the training set were first one-hot encoded, and synthetic samples for the minority class (PPCs = Yes) were generated in this feature space via k-nearest-neighbor interpolation.SMOTE was implemented using the R package smotefamily, with the number of neighbors set to K = 5 and the oversampling multiplier set to dup_size = 2. Subsequently, the KNN model was retrained on the augmented training set.To avoid information leakage and overestimation of performance, no resampling was performed on the test set, which consistently retained the original class distribution for independent evaluation.

To enhance the transparency of the feature-selection rationale and to assess whether linear screening might miss nonlinear information, we compared an 11-variable KNN model with a full 20-variable KNN model under the same training/test split and identical preprocessing and hyperparameter-tuning strategies.In the independent test set, we reported the AUC, sensitivity/specificity, calibration (calibration curves/Brier score), and net benefit from DCA.

Ultimately, the KNN model was identified as the most effective predictive model through comprehensive evaluation, and the SHAP method was utilized for feature interpretation of the optimal model. Feature ranking was determined by calculating SHAP values and ranking features according to their absolute values.

### 2.5 Statistical analysis

Continuous data were statistically analyzed using the t-test and are expressed as mean ± standard deviation. Categorical data were analyzed using the Chi-squared test and are presented as frequency (percentage). This study utilized R version 4.4.2 throughout the analysis.

## 3 Result

### 3.1 Patient characteristics

**Figure 1:**
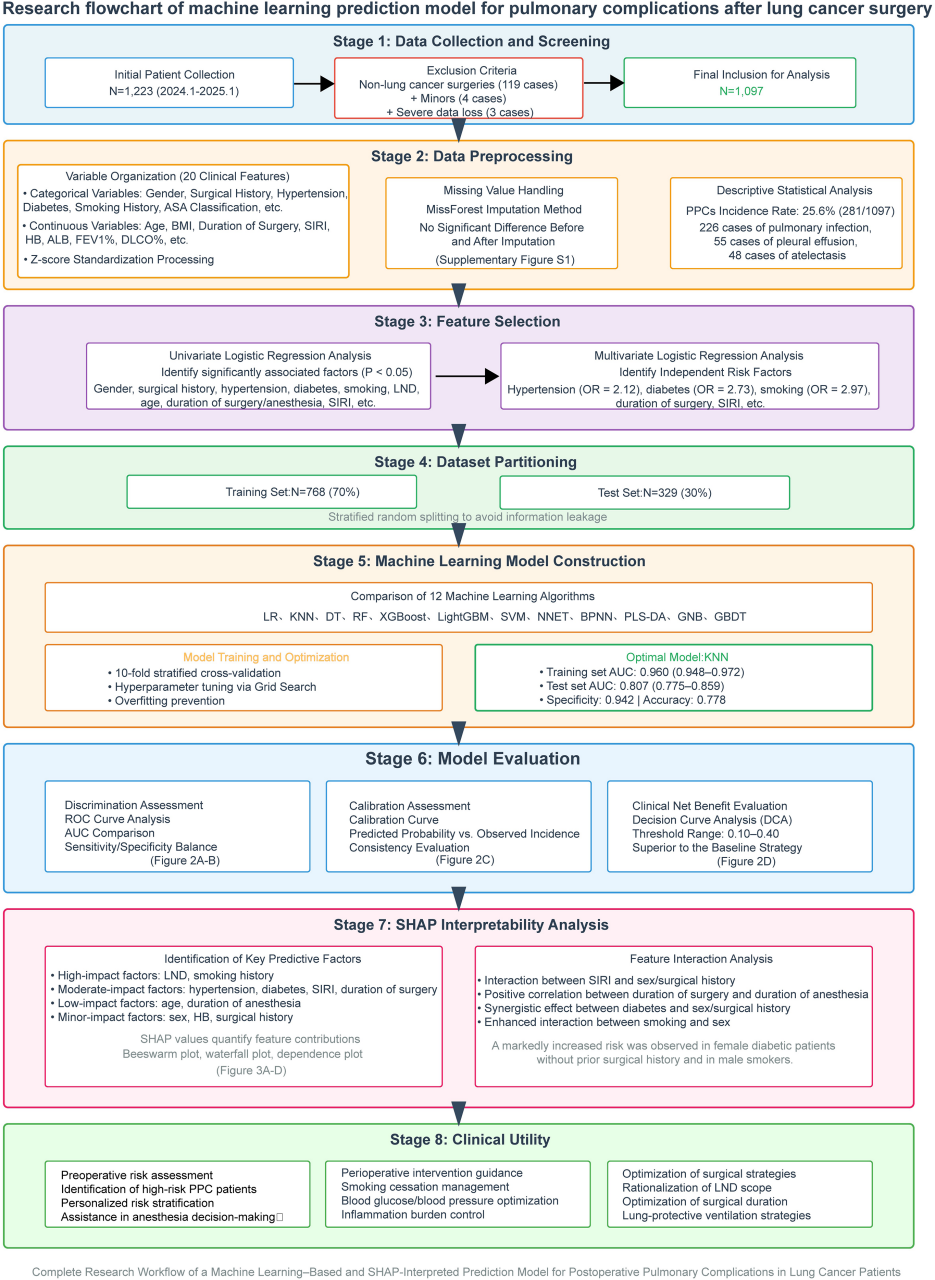
Flowchart.

This study included 1, 097 postoperative lung cancer patients, among whom 281 (25.6%) experienced PPCs. These complications included 226 cases of pulmonary infection, 55 cases of pleural effusion, and 48 cases of atelectasis, with a few cases presenting combined pneumothorax and chylothorax. The study design details are presented in **Figure 1**. Missing data were handled using MissForest for nonparametric iterative imputation, and most variables had no missing values.Among key pulmonary function indices, FEV1% was missing in 38 cases (3.46%) and DLCO% was missing in 44 cases (4.01%); the missingness rates of all other continuous variables were low (maximum 0.09%) (**Supplementary Table 3**).Comparison of the distributions of key variables before and after imputation showed no clinically meaningful systematic changes (**Supplementary Figure 1**).Therefore, subsequent analyses were conducted using the imputed dataset.The stratified box plot (**Supplementary Figure 2**) revealed that the PPCs group had longer surgical and anesthesia times, higher inflammatory burden (noted by increased systemic inflammatory response index (SIRI) and a higher dispersion, and a slight elevation in WBC count), and poorer nutritional and hematological status (HB, ALB) as well as reduced lung function reserve (FEV1%, DLCO%), consistent with trends observed in **Table 1**. The distribution of categorical variables (**Supplementary Figure 3**) indicated that the sample predominantly consisted of females and patients classified as ASA II, with most lacking a history of previous surgeries and cardiovascular metabolic comorbidities, and a certain proportion being smokers; the surgical side was slightly more often on the right, with a comparable rate of LND. This baseline composition provides necessary clinical context and comparability for model performance and interpretative analysis.

The differences in the distribution of categorical variables such as gender, previous surgical history (PSH), hypertension, diabetes, smoking history, LND, and ASA classification between the two patient groups were statistically significant (P<0.05, **Table 1**). Specifically, patients who developed PPCs were predominantly male (53.4% vs. 37.4%) and had a significantly higher proportion of comorbidities such as hypertension (26.3% vs. 13.6%), diabetes (23.5% vs. 10.5%), and a history of smoking (46.3% vs. 19.9%). Additionally, the proportion of patients undergoing LND during surgery was markedly higher in the PPCs group (63.7% vs. 43.1%), and those with higher ASA classifications (III–IV) had an increased risk of complications. In contrast, there was no significant difference in the distribution of surgical sites and the presence of coronary artery disease between the two groups. Overall, patients in the PPCs group were older, had longer anesthesia and surgical durations, exhibited higher SIRI levels, and had slightly lower HB levels, indicating that the occurrence of PPCs is closely related to the patients’ baseline conditions and perioperative stress responses.

### 3.2 Development of a logistic regression nomogram based on key feature selection

Through univariate and multivariate LR analyses, significant factors influencing the occurrence of PPCs were identified. The univariate analysis indicated that gender, PSH, hypertension, diabetes, smoking history, LND, and anesthesia duration were significantly associated with the occurrence of PPCs (**Table 2**). In the multivariate regression analysis, hypertension, diabetes, smoking history, surgical duration, and the SIRI were identified as independent risk factors. LND is an intraoperative procedural variable, and its regression coefficient and OR primarily reflect predictive association and may encode information on clinical need, disease burden, and operative complexity; therefore, this variable should not be interpreted causally. Specifically, hypertension (OR = 2.12, p<0.001), diabetes (OR = 2.73, p<0.001), smoking history (OR = 2.97, p<0.001), and longer surgical duration (OR = 1.05, p<0.001) were found to significantly increase the risk of PPCs (**Table 2**).

**Figure 2:**
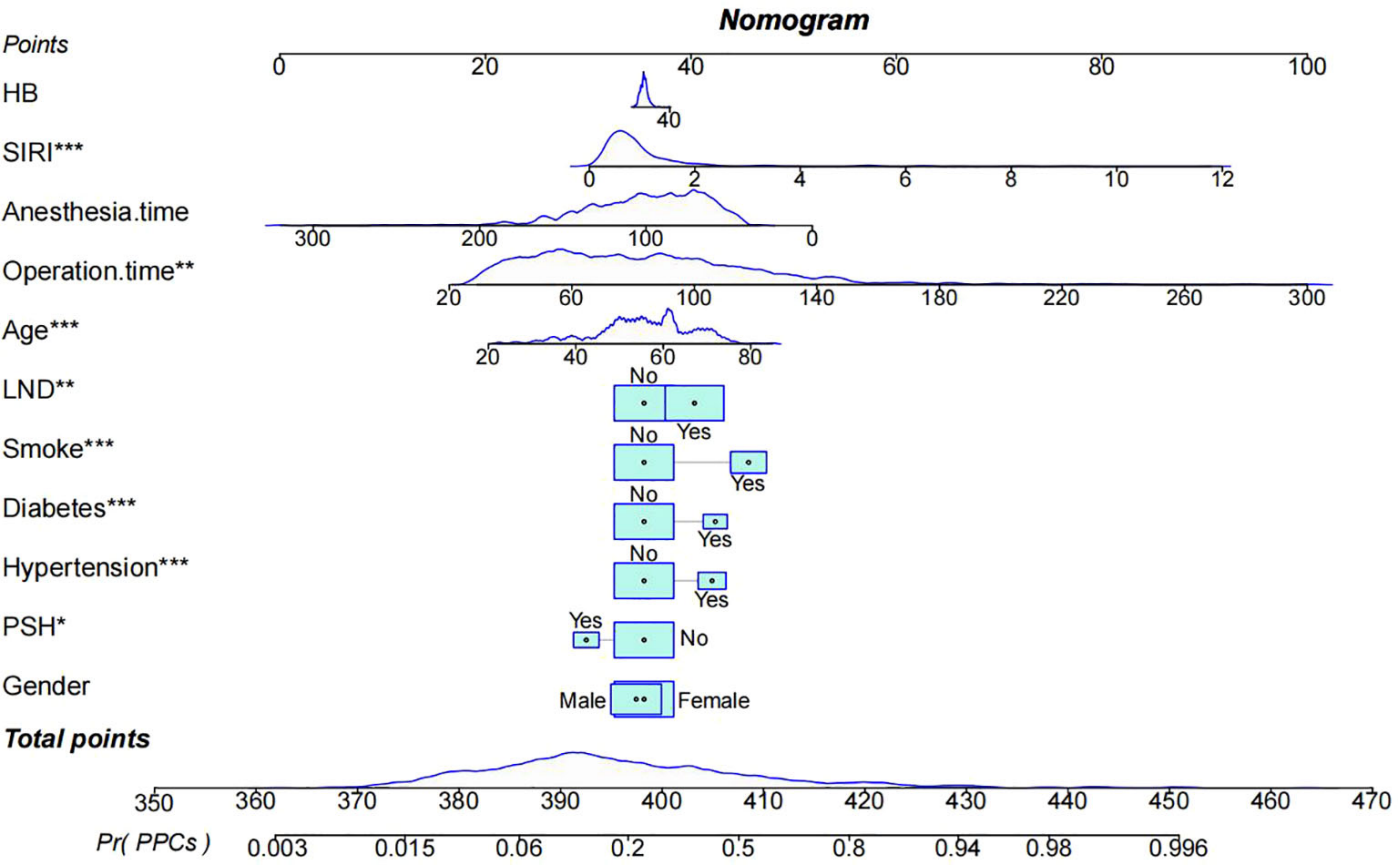
LR model nomogram.

Based on the results of the univariate and multivariate LR analyses, a nomogram was constructed to predict PPCs in lung cancer patients. As an intraoperative procedural variable, LND was included primarily to improve the predictive performance for perioperative PPCs, and it should not be clinically interpreted as a directly modifiable preoperative risk factor.The nomogram included eleven variables: HB, SIRI, duration of surgery/anesthesia, age, LND, smoking history, diabetes, hypertension, PSH, and gender. Each variable was assigned a score, and the total score represents the likelihood of PPCs. The nomogram illustrates that smoking history, diabetes, hypertension, SIRI, and LND are significant contributors to the prediction (**Figure 2**).

### 3.3 Machine learning model building

To ensure the scientific progression of the analysis and enhance its clinical interpretability, we initially constructed a baseline model using multifactor LR (Baseline-LR). This model serves as a referential framework, facilitating the interpretation of the direction and magnitude of variable effects. Building upon this foundation, we systematically compared various machine learning algorithms to assess their potential to provide incremental value over Baseline-LR, particularly in modeling nonlinear relationships and feature interactions.

In an effort to construct an accurate predictive model for lung cancer, this study utilized a comparative analysis of 12 classical machine learning algorithms: LR, KNN, DT, RF, XGBoost (XGBoost), Light Gradient Boosting Machine (LightGBM), Support Vector Machine (SVM), Neural Network (NNET), Backpropagation Neural Network (BPNN), Partial Least Squares Discriminant Analysis (PLS-DA), Gaussian Naive Bayes (GNB), and Gradient Boosting Decision Tree (GBDT). All datasets were randomly divided into a training set (n=768) and a testing set (n=329), with the implementation of a 10-fold CV method to mitigate the risk of overfitting. Hyperparameter tuning for all models was meticulously conducted using the Grid Search method to ensure that each algorithm was optimized under the best possible settings (**Table 3**).

**Table 3:** Model evaluation metrics for the training set and test set.

ML model	AUC (95%CI)	Accuracy	NPV	PPV	Sensitivity	Specificity
Logit						
Train	0.844 (0.813-0.876)	0.824	0.843	0.73	0.477	0.941
Test	0.779 (0.720-0.837)	0.772	0.792	0.644	0.33	0.934
KNN						
Train	0.960 (0.948-0.972)	0.889	0.883	0.922	0.611	0.983
Test	0.807 (0.775-0.859)	0.778	0.794	0.674	0.33	0.942
DT						
Train	0.841 (0.805-0.877)	0.867	0.878	0.818	0.606	0.955
Test	0.712 (0.647-0.777)	0.747	0.788	0.545	0.341	0.896
RF						
Train	1.000 (1.000-1.000)	0.990	1.000	0.960	1	0.986
Test	0.791 (0.738-0.843)	0.766	0.831	0.568	0.522	0.855
XGBoost						
Train	0.962 (0.949-0.975)	0.904	0.897	0.934	0.663	0.984
Test	0.797 (0.743-0.851)	0.793	0.810	0.700	0.398	0.938
LightGBM						
Train	0.987 (0.981-0.992)	0.922	0.904	0.974	0.774	0.994
Test	0.802 (0.749-0.854)	0.757	0.822	0.580	0.543	0.843
SVM						
Train	0.919 (0.896-0.943)	0.850	0.850	0.850	0.656	0.944
Test	0.790 (0.736-0.845)	0.766	0.784	0.516	0.548	0.883
NNET						
Train	0.909 (0.879-0.938)	0.895	0.939	0.762	0.808	0.922
Test	0.761 (0.701-0.820)	0.742	0.846	0.455	0.519	0.810
BPNN						
Train	0.910 (0.878-0.942)	0.952	0.974	0.886	0.919	0.962
Test	0.732 (0.669-0.796)	0.757	0.855	0.489	0.551	0.821
PLS-DA						
Train	0.866 (0.838-0.894)	0.828	0.963	0.425	0.796	0.833
Test	0.795 (0.740-0.851)	0.772	0.942	0.307	0.659	0.788
GNB						
Train	0.818 (0.783-0.852)	0.807	0.920	0.472	0.664	0.838
Test	0.759 (0.698-0.819)	0.763	0.909	0.364	0.523	0.796
GBDT						
Train	0.937 (0.920-0.955)	0.876	0.965	0.611	0.855	0.881
Test	0.788 (0.735-0.841)	0.769	0.913	0.375	0.611	0.800

Among all the machine learning models compared, the KNN model achieved an AUC of 0.807 (95% CI 0.775–0.859) on the test set, significantly outperforming other models, such as XGBoost (AUC 0.797) and LightGBM (AUC 0.802). Although the KNN model slightly underperforms in terms of AUC compared to some models, it demonstrates a considerable advantage in balancing sensitivity and specificity, excelling notably in specificity, which reached 0.942.

**Figure 3:**
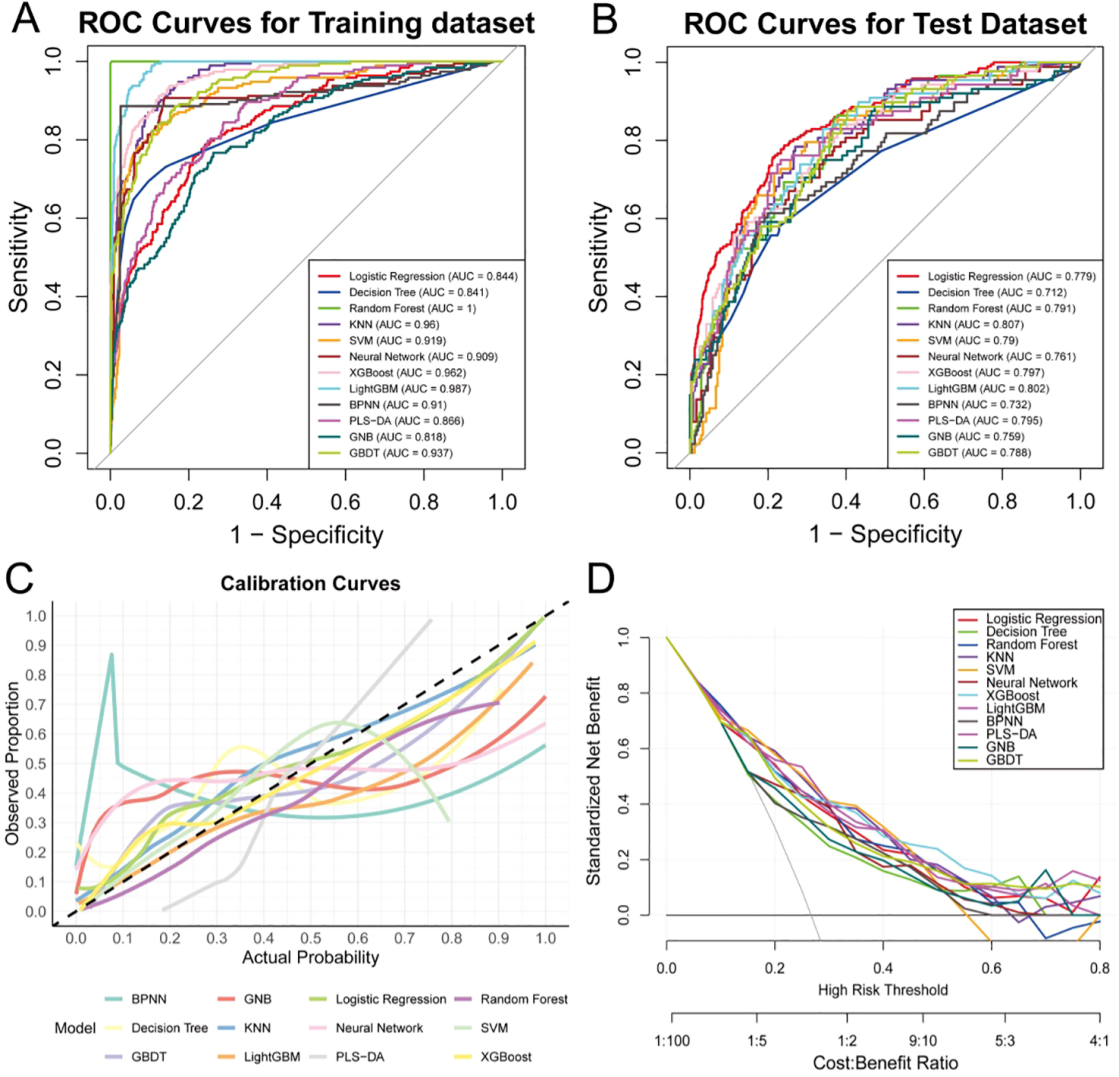
Machine learning model curves. **(A, B)** ROC curves for the training and test sets. **(C)** Calibration curves. **(D)** DCA curves.

The research data were stratified and randomly divided into training and testing sets in a 7:3 ratio. All model development and hyperparameter tuning were conducted using CV on the training set, whereas the testing set was used solely for a one-time independent evaluation. Within this framework, we assessed the performance of various models based on the ROC curve (**Figures 3A, B**). We observed that the AUC of the KNN model on the training set was 0.960 (95% CI 0.948–0.972), and on the testing set, it was 0.807 (95% CI 0.775–0.859), indicating that the KNN model possesses high diagnostic capability, particularly demonstrating robust performance in the training set. The standard curve indicates that the KNN model aligns closely with the ideal diagonal line within the probability range of 0.2–0.6, suggesting that the KNN model exhibits good predictive calibration in practical applications (**Figure 3C**).

DCA showed that within a threshold probability range of 0.10–0.40, the model provided a higher net benefit than either the treat-all or treat-none strategies.Accordingly, we recommend an operating threshold as follows: patients with a model-predicted PPCs probability of ≥ 0.30 should be classified as high risk, indicating that perioperative management should be escalated (**Figure 3D**). These results suggest that the KNN model can provide significant net benefits for clinicians in perioperative risk stratification and intervention decision-making, thereby demonstrating strong clinical applicability.

In addition, we compared the 11-variable KNN model with the full 20-variable feature model.The full-feature model exhibited perfect fitting in the training set but provided no gain in the test set, with the AUC decreasing from 0.807 to 0.787, sensitivity increased from 0.33 to 0.42, whereas specificity decreased from 0.942 to 0.917 (**Supplementary Table 2**).The calibration curve and DCA net-benefit curve of the full-feature model were overall similar to those of the 11-variable model (**Supplementary Figure 4**).Considering generalization risk, model complexity, and the clinical burden of data collection, we retained the 11-variable model as the primary analysis in the main text, and placed the full-feature results in the **Supplementary Materials**.

Under the default threshold (cutoff = 0.5), the KNN model achieved a sensitivity of 0.33 in the testing set, suggesting that approximately 67% of patients with PPCs could be missed, which limits its utility for clinical screening.Given that false negatives carry a higher cost in early warning, we lowered the threshold to cutoff = 0.3 and evaluated performance with this fixed threshold in an independent testing set.After threshold adjustment, testing set sensitivity increased from 0.33 to 0.591, with specificity of 0.834, indicating a marked reduction in missed cases at an acceptable level of specificity.To address class imbalance, we applied SMOTE to the training set and retrained the KNN model.After SMOTE, the model achieved a testing set sensitivity of 0.539 and specificity of 0.830 (**Table 4**).

**Table 4:** KNN model threshold adjustment and SMOTE processing.

ML model	AUC (95%CI)	Accuracy	NPV	PPV	Sensitivity	Specificity
Model_knn						
Train	0.960 (0.948-0.972)	0.889	0.883	0.922	0.611	0.983
Test	0.807 (0.775-0.859)	0.778	0.794	0.674	0.33	0.942
Cutoff=0.3						
Train	0.960 (0.948-0.972)	0.893	0.751	0.881	0.860	0.904
Test	0.807 (0.775-0.859)	0.769	0.848	0.565	0.591	0.834
SMOTE						
Train	1.000 (1.000-1.000)	0.995	1.000	0.990	1.000	0.990
Test	0.732 (0.670-0.794)	0.754	0.833	0.539	0.546	0.830

### 3.4 Shapley additive explanations

**Figure 4:**
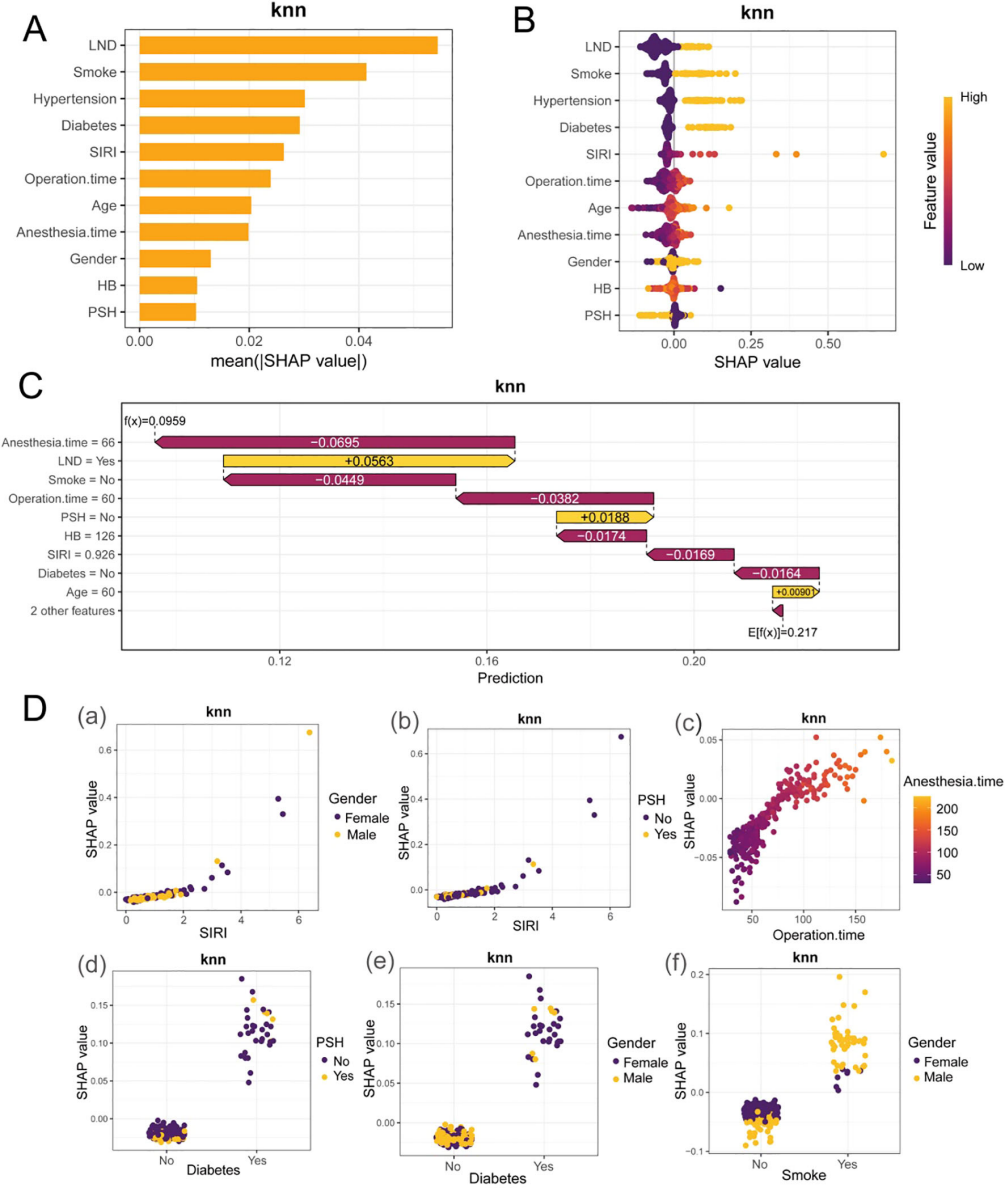
Shapley additive explanations. **(A, B)** SHAP analysis important feature distribution bar chart and beeswarm plot. **(C)** Single sample feature force plot. **(D)** a-b. Dual feature dependence plot of SIRI with gender and PSH; c. Dual feature dependence plot of surgical duration and anesthesia time; d-e. Dual feature dependence plot of diabetes with gender and PSH; f. Dual feature dependence plot of smoking with gender.

SHAP analysis identifies the primary predictive factors of the KNN model as LND and smoking, followed by hypertension, diabetes, SIRI, and surgical duration. Age and anesthesia time contribute moderately to the model, whereas gender, HB, and PSH have minimal impacts. Among the above factors, LND is an intraoperative procedural variable, and SHAP reflects its importance and direction of association in the model’s predictions, it may partially encode information on clinical need, disease burden, and operative complexity, and therefore should not be interpreted causally.The beeswarm plot illustrates that high values of risk factors correlate with a concentration of positive SHAP values, indicating an increase in risk, whereas protective factors show a negative contribution. The waterfall chart further demonstrates that prolonged anesthesia time and extensive LND significantly elevate the predicted probability of adverse outcomes, while non-smoking status and shorter surgical durations are associated with reduced risk. Dependence analysis reveals a monotonically increasing relationship between SIRI and SHAP values, indicating that as SIRI increases, so does its contribution to risk. Additionally, the risk associated with extended surgical duration is synergistically amplified by prolonged anesthesia time. In contrast, diabetes and smoking exhibit a stepwise enhancement in their effects, while gender and PSH only demonstrate mild modulation of risk. Collectively, LND, smoking, hypertension, diabetes, SIRI, surgical duration, age, and anesthesia time are critical in driving risk assessments, whereas the independent contributions of HB, gender, and PSH are limited. Moreover, the analysis of interactions among clinical features reveals that SHAP values for SIRI are predominantly influenced by female patients without previous surgical history, as shown in **Figure 4D(a-b)**. There exists a linear positive correlation between surgical duration and anesthesia time; an increase in surgical duration not only elevates SHAP values but also leads the model to predict a higher probability of PPCs. The model’s predictive probability is significantly enhanced in female diabetic patients without prior surgical history (**Figure 4D(d-e)**) and in smoking male patients (**Figure 4D(f)**).

To explore the relationship between operative time and the effect of SIRI, we plotted the SHAP dependence plot for SIRI in the KNN model and colored the points by operation time (**Supplementary Figure 5**).The results showed that when SIRI was low (approximately < 2), the SHAP values were generally close to 0 and were minimally influenced by operative time.When SIRI increased (approximately > 3), the SHAP values rose markedly, and at comparable SIRI levels, individuals with longer operative times corresponded to higher SHAP values.

## 4 Discussion

In light of the limitations of existing models, this study conducted a systematic comparison of twelve machine learning models using real-world data from a single center, beginning with the decision-making scenarios typical of anesthesiologists. Discriminative and calibration performances were evaluated under a consistent data and process framework, employing stratified CV and a held-out test set. We incorporated DCA to quantify the net benefits at various clinical thresholds and utilized SHAP values to enhance interpretability at both global and individual levels. The findings demonstrated that the KNN model provided the most effective performance in the test set, exhibiting superior discriminative ability while maintaining stable calibration. The DCA confirmed its significant net benefit within clinically relevant threshold ranges. In early warning for PPCs, false negatives imply that high-risk patients are not identified in a timely manner, and their potential harm typically outweighs that of false positives.Therefore, model deployment should not mechanically adopt the default threshold of 0.5, instead, the threshold should be set in accordance with clinical objectives and resource constraints.In this study, the KNN model achieved a test-set sensitivity of 0.33 at cutoff = 0.5.After lowering the threshold to 0.3 and evaluating it as a fixed cutoff, sensitivity increased to 0.591 while specificity remained 0.834, suggesting that reducing missed cases at an acceptable false-alarm level better aligns with clinical use.In addition, given the class imbalance with a PPCs incidence of approximately 25.6%, we performed a SMOTE sensitivity analysis in the training set, while keeping the test set at its original distribution to avoid information leakage.After SMOTE, test-set sensitivity was 0.539 and specificity was 0.830, but the AUC decreased despite excellent training-set performance, indicating that oversampling may introduce overfitting risk and should be further weighed under external validation or within a cost-sensitive learning framework.To assess whether linear screening might omit strongly predictive nonlinear features, we compared the 11-variable KNN model with the full 20-variable KNN model.Although the full-feature model performed better in the training set, it was not overall superior to the 11-variable model in the testing set, and differences in calibration and DCA were limited, considering generalization risk and the clinical burden of data collection, the 11-variable model was retained as the primary model in the main text.SHAP analysis identified LND, smoking, hypertension, diabetes, SIRI, surgical duration, age, and anesthesia duration as critical factors influencing predictions, offering actionable insights for intervention strategies such as smoking cessation management, optimization of blood glucose and blood pressure levels, control of inflammatory load, and management of perioperative processes.In this study, LND was recorded only as a binary indicator of whether it was performed, and the data could not distinguish differences in extent (e.g., systematic mediastinal dissection vs sampling), therefore, its risk weight should not be interpreted as implying that a more extensive dissection is associated with a higher or lower risk.In addition, the observed higher risk of PPCs among patients undergoing LND should be interpreted in light of potential confounding by indication.More extensive dissection may reflect a higher tumor burden, more complex anatomical dissection, and longer durations of surgery and one-lung ventilation.This study corroborates previous research identifying LND, smoking, surgical duration, anesthesia duration, and age as significant risk factors for PPCs (3, 19, 20). Elderly patients face an elevated risk of PPCs due to physiological decline and a greater prevalence of comorbidities such as COPD, cardiovascular, and metabolic disorders (21). Chronic smoking aggravates the deterioration of lung function through inflammation of the small airways, thereby increasing susceptibility to complications (3). An extended surgical duration significantly raises the risk of PPCs, with patients undergoing LND facing approximately twice the risk compared to those without such procedures.

Although anesthesia duration is positively correlated in univariate analyses, it exhibits a negative coefficient in multivariate models due to high collinearity with surgical duration, yet it can still serve as a proxy for “duration-type exposure.” Mechanistically, prolonged surgical duration increases risks associated with one-lung ventilation, operative trauma, and systemic inflammatory response, accompanied by fluid accumulation, hypothermia, and cumulative anesthetic dosage. These factors collectively contribute to alveolar shear stress, oxidative stress, and limited lung re-expansion, leading to atelectasis, infection, and hypoxia (22). Anesthesia duration reflects the ‘dose-time’ effect of anesthetic management factors, including mechanical ventilation, intrapulmonary shunting, temperature, and acid-base balance (23, 24). An expansion in the scope of LND can compromise the blood supply and lymphatic drainage at the bronchial stumps, causing edema, secretion retention, and damage to the mucosal barrier. Concurrently, it heightens the risk of traction and nerve damage, impairing cough and sputum clearance functions (25). LND is often correlated with extended one-lung ventilation time and overall surgical duration, which collectively increase the incidence of PPCs (26). However, these inferences require validation within a more rigorous causal-inference framework; therefore, clinical decision-making should integrate patients’ baseline risk and perioperative management while adhering to oncologic principles. This includes limiting the extent of LND in accordance with oncologic requirements, optimizing surgical processes, and strengthening lung-protective ventilation as well as temperature, fluid, and pain management to mitigate the risk of PPCs.

Hypertension and diabetes, recognized as independent high-risk factors, substantially elevate the risk of PPCs. In hypertensive patients, chronic endothelial dysfunction and small vessel remodeling contribute to diminished pulmonary capillary permeability and compromised regulation of tissue perfusion. During the perioperative period, these patients demonstrate heightened sensitivity to volume load and hemodynamic fluctuations. The concomitant challenges of one-lung ventilation and surgical stress predispose them to atelectasis, pulmonary edema, and infection (27). In individuals with diabetes, variability in blood glucose levels undermines neutrophil and macrophage functionality, impedes phagocytosis and chemotaxis, and delays wound healing. Additionally, microvascular lesions and abnormalities in collagen metabolism impair the healing of bronchial stumps and lung parenchyma, thus heightening the risk of postoperative pneumonia and respiratory failure (28).

SIRI is robustly associated with PPCs, as evidenced by both univariate and multivariate regression analyses, asserting its role as an independent risk factor. Given the dynamic nature of SIRI and its susceptibility to the timing of measurement and perioperative stress status, we restricted the value window to within 7 days before surgery or the most recent preoperative test to reduce heterogeneity arising from differences in blood-draw timing and to facilitate perioperative risk stratification.The results suggest a potential synergistic amplification effect between SIRI and operative duration, whereby a high inflammatory state combined with longer surgical/anesthetic exposure may further increase the risk of PPCs. A high SIRI, indicative of an inflammatory-immune imbalance, is characterized by elevated neutrophils/monocytes and diminished lymphocytes. This imbalance leads to innate immunity overactivation, which releases inflammatory mediators that increase the permeability of the alveolar-capillary barrier. Under the conditions of one-lung ventilation and surgical trauma, such alterations may precipitate atelectasis, exudation, and hypoxia. Concurrently, lymphopenia compromises adaptive immune defenses, diminishes pathogen clearance capacity, and amplifies infection risk (29). Furthermore, SIRI encapsulates the chronic low-grade inflammation associated with smoking, metabolic comorbidities, and aging, maintaining its independent predictive power even after adjusting for baseline disparities. This suggests that SIRI not only serves as a surrogate marker for comorbidity burden but also quantifies the bidirectional imbalance between perioperative inflammatory activity and immune suppression. Patients with elevated SIRI are more susceptible to PPCs, progressing along the pathway of “inflammatory amplification-ventilation/diffusion limitation-susceptibility to infection, “ thereby demonstrating significant clinical prognostic value.SHAP dependence plots can provide clues regarding potential nonlinearity and interactions; however, this finding is exploratory, and its statistical significance still requires further confirmation through explicit interaction-term modeling or stratified analyses.

In addition, **Figure 4D** suggests that some variables may exhibit nonlinearity and interactions. It should be emphasized that SHAP explains feature contributions and associations under the current data distribution, and it does not constitute causal evidence that can directly guide interventions.Therefore, these synergistic patterns are better suited to identifying risk heterogeneity and high-risk combinations for risk communication and decision support, rather than being mechanically translated into fixed escalation rules for interventions.We recommend using the model-derived individualized risk probability as the trigger for clinical pathways, with risk stratification implemented via prespecified thresholds (e.g., standard vs intensified management).The key relevance of interaction effects lies in their potential to push the overall risk across a threshold, thereby placing a patient into a higher-intensity perioperative management pathway.

Moreover, this study identified that gender exhibited a higher risk of PPCs in univariate analysis; however, this association dissipated after multivariate adjustment, likely due to confounding bias. The principal determinants of risk include age, LND, duration of surgery, and SIRI, among others. If these modifiable factors are disproportionately distributed across genders, they may create observable gender disparities. For example, the independent predictor of a smoking history also presents an uneven gender distribution that might account for this observed discrepancy. Consequently, the noted gender differences likely reflect a bias in the distribution of risk exposure rather than an inherent biological effect.

The analysis of HB suggests a protective role; however, within a multifactorial model, it fails to demonstrate independent predictive significance, suggesting a nonlinear relationship. Anemia diminishes oxygen delivery and heightens the likelihood of transfusions, while elevated HB levels increase blood viscosity; both conditions amplify the risk of PPCs (30). It is advised to rectify anemia, abstain from unnecessary transfusions, cease smoking, and enhance ventilation strategies. A history of prior surgeries correlates with a diminished risk of PPCs, indicative of selection bias and superior perioperative preparation: such patients often present with more favorable surgical indications, exhibit greater adherence to Enhanced Recovery After Surgery (ERAS) protocols, and benefit from comprehensive risk assessments and interventions (31).

To preclude information leakage, this study exclusively incorporated preoperative and intraoperative variables, with outcomes confined to PPCs occurring within seven days. Because this model was developed for perioperative risk prediction, intraoperative procedural variables (e.g., LND and surgical/anesthetic duration) were included to improve predictive performance. Therefore, the model is not intended for purely preoperative decision support. Clinically, the duration of chest drainage and the extent of antibiotic administration are significant indicators of risk; nevertheless, both are subject to the influences of disease severity, intraoperative occurrences, and early PPCs. Extended drainage may exacerbate pain and impede sputum elimination, whereas excessive use of antibiotics may disrupt the microbiome and adversely impact the gut-lung axis (32, 33). Direct inclusion of these variables in a static baseline model would introduce confounding bias; therefore, they were excluded from this study. Future research should focus on developing a dynamic early warning model within a multicenter, large-sample framework to distinguish predictive factors from disease markers, thereby providing a foundation for bedside interventions.

The DCA threshold reflects a trade-off between the risk of missed diagnoses and the costs of overtreatment, and there is no universal cut-off.The proposed threshold of 0.30 should be regarded as a pragmatic operating threshold to trigger further assessment and optimization, rather than a rigid standard that replaces clinical judgment.For elective surgery, shared decision-making is recommended: when the predicted probability is ≥ 0.30 and reversible factors are present (e.g., inadequate control of bronchospasm, recent respiratory tract infection, ongoing smoking, or poor nutritional status/low albumin), short-term optimization followed by reassessment may be considered after multidisciplinary evaluation.However, whether to postpone surgery should still be determined in conjunction with factors such as tumor stage/surgical window, symptom severity, and resource availability.With future external validation and implementation studies, stratified thresholds and corresponding pathway-based intervention bundles could be further refined to improve feasibility and consistency.

In this study, the incidence of PPCs was observed to be 25.7%, which exceeds the reported average of 18.4% found in the literature (3), primarily attributable to variations in diagnostic criteria. The definition of PPCs lacks uniformity, and existing scoring systems such as the Surgical Trauma Score (STS) or External Trauma Score (EST), Surgical Pulmonary Embolism Score (STEP), and European Perioperative Cardiac Outcome (EPCO) are primarily designed for the general surgical population, rendering them less applicable to patients with lung cancer (5, 34, 35). This study utilized the STEP criteria and the CDC pneumonia diagnostic standards, necessitating the integration of imaging and clinical characteristics. However, early postoperative imaging alterations, elevated white blood cell counts, and transient respiratory symptoms are typical physiological responses that may readily be confounded with PPCs, leading to an augmented rate of misdiagnosis.

The current model predicts the risk of PPCs within 7 days after surgery using preoperative and intraoperative variables, and is therefore applicable to perioperative risk stratification.However, because some PPCs develop gradually over several postoperative days, future work could extend the model toward dynamic monitoring and early warning.Specifically, postoperative process data from 0–24 hours and beyond (e.g., longitudinal inflammatory markers such as leukocyte count/CRP, chest drainage volume and trends, and parameters of oxygenation and respiratory support) could be incorporated into the existing framework to enable stage-wise risk updating.

This study acknowledges several limitations: (1) The diagnostic criteria for PPCs are not consistent, and the adoption of STEP and CDC standards may lead to an overestimation of incidence due to confounding effects from postoperative physiological responses and complications; (2) The single-center retrospective design constrains external validity; (3) With MissForest imputation and missingness rates of < 5% for key pulmonary function indices, the risk of substantial systematic bias introduced by imputation is relatively limited. If the missingness is not MNAR, validation in an external cohort is still required; (4) The 10-fold CV of homogenous data poses a risk of overfitting, and its generalizability requires validation in independent cohorts; (5) External factors such as lifestyle, environment, and genetics were not accounted for; (6) Although SHAP enhances interpretability, uncertainties remain concerning complex interaction effects. Future research should prioritize multi-center prospective studies to enhance validation.(7) Because SIRI is influenced by multiple factors and exhibits time dependence, further multicenter and prospective studies are needed for validation.

## 5 Conclusion

This study has successfully developed a predictive model for PPCs in lung cancer patients by integrating machine learning algorithms with the SHAP interpretation method. The model proficiently forecasts the risk of PPCs, utilizing preoperative clinical characteristics, laboratory indicators, and surgical information. Consequently, it furnishes clinicians with personalized guidelines for perioperative care and anesthetic interventions.

## Data Availability

The original contributions presented in the study are included in the article/**Supplementary Materials**. Further inquiries can be directed to the corresponding authors.

## References

[1] Bray F Laversanne M Sung H Ferlay J Siegel RL Soerjomataram I . Global cancer statistics 2022: GLOBOCAN estimates of incidence and mortality worldwide for 36 cancers in 185 countries. CA Cancer J Clin. (2024) 74:229–63. doi: 10.3322/caac.21834, PMID: 38572751

[2] Kidane B Bott M Spicer J Backhus L Chaft J Chudgar N . The American Association for Thoracic Surgery (AATS) 2023 Expert Consensus Document: Staging and multidisciplinary management of patients with early-stage non-small cell lung cancer. J Thorac Cardiovasc Surg. (2023) 166:637–54. doi: 10.1016/j.jtcvs.2023.04.039, PMID: 37306641

[3] Deng T Song J Tuo J Wang Y Li J Ping Suen LK . Incidence and risk factors of pulmonary complications after lung cancer surgery: A systematic review and meta-analysis. Heliyon. (2024) 10:e32821. doi: 10.1016/j.heliyon.2024.e32821, PMID: 38975138 PMC11226845

[4] Chen S Deng T Yang Q Li J Shen J Luo X . Development and validation of an explainable machine learning model for predicting postoperative pulmonary complications after lung cancer surgery: a machine learning study. EClinicalMedicine. (2025) 86:103386. doi: 10.1016/j.eclinm.2025.103386, PMID: 40791887 PMC12337024

[5] Abbott TEF Fowler AJ Pelosi P Gama de Abreu M Møller AM Canet J . A systematic review and consensus definitions for standardised end-points in perioperative medicine: pulmonary complications. Br J Anaesth. (2018) 120:1066–79. doi: 10.1016/j.bja.2018.02.007, PMID: 29661384

[6] Miskovic A Lumb AB . Postoperative pulmonary complications. Br J Anaesth. (2017) 118:317–34. doi: 10.1093/bja/aex002, PMID: 28186222

[7] Huang Z Han Y Zhuang H Jiang J Zhou C Yu H . Prediction models for postoperative pulmonary complications: a systematic review and meta-analysis. Br J Anaesth. (2025) 135:1415–27. doi: 10.1016/j.bja.2025.04.025, PMID: 40473567 PMC12597335

[8] Gupta H Gupta PK Fang X Miller WJ Cemaj S Forse RA . Development and validation of a risk calculator predicting postoperative respiratory failure. Chest. (2011) 140:1207–15. doi: 10.1378/chest.11-0466, PMID: 21757571

[9] Qaseem A Snow V Fitterman N Hornbake ER Lawrence VA Smetana GW . Risk assessment for and strategies to reduce perioperative pulmonary complications for patients undergoing noncardiothoracic surgery: a guideline from the American College of Physicians. Ann Intern Med. (2006) 144:575–80. doi: 10.7326/0003-4819-144-8-200604180-00008, PMID: 16618955

[10] Wang B Chen Z Zhao R Zhang L Zhang Y . Development and validation of a nomogram to predict postoperative pulmonary complications following thoracoscopic surgery. PeerJ. (2021) 9:e12366. doi: 10.7717/peerj.12366, PMID: 34760381 PMC8572520

[11] Khanna AK Kelava M Ahuja S Makarova N Liang C Tanner D . A nomogram to predict postoperative pulmonary complications after cardiothoracic surgery. J Thorac Cardiovasc Surg. (2023) 165:2134–46. doi: 10.1016/j.jtcvs.2021.08.034, PMID: 34689983

[12] Kong X Yuan Q Wang H Liu M Niu Z Lu J . Risk factors and predictive model for postoperative pulmonary complications following pancreaticoduodenectomy: a retrospective cohort study. Langenbecks Arch Surg. (2025) 410:300. doi: 10.1007/s00423-025-03681-0, PMID: 41099886 PMC12532676

[13] Wang Q Li Y Zhao K Ping Z Zhang J Zhou J . A risk prediction nomogram model for postoperative pulmonary complications in children aged 0–6 years. Risk Manag Healthc Pol. (2025) 18:1085–97. doi: 10.2147/RMHP.S507147, PMID: 40177648 PMC11963797

[14] Susnjak T Griffin E . Towards clinical prediction with transparency: An explainable AI approach to survival modelling in residential aged care. Comput Methods Programs Biomed. (2025) 263:108653. doi: 10.1016/j.cmpb.2025.108653, PMID: 39970690

[15] Smith LA Oakden-Rayner L Bird A Zeng M To MS Mukherjee S . Machine learning and deep learning predictive models for long-term prognosis in patients with chronic obstructive pulmonary disease: a systematic review and meta-analysis. Lancet Digit Health. (2023) 5:e872–e81. doi: 10.1016/S2589-7500(23)00177-2, PMID: 38000872

[16] Tang D Liang F Gu X Jin Y Hu X Liu F . Exploration and analysis of risk factors for coronary artery disease with type 2 diabetes based on SHAP explainable machine learning algorithm. Sci Rep. (2025) 15:29521. doi: 10.1038/s41598-025-11142-3, PMID: 40796917 PMC12344076

[17] Yuan Y Zheng F Yao J Zhou K Yang J Liu X . Personalized prediction for recurrence of cystitis glandularis: insights from SHAP and machine learning models. Transl Androl Urol. (2025) 14:808–19. doi: 10.21037/tau-2024-665, PMID: 40226087 PMC11986474

[18] Garutti I de la Gala F Piñeiro P Rancan L Vara E Reyes A . Usefulness of combining clinical and biochemical parameters for prediction of postoperative pulmonary complications after lung resection surgery. J Clin Monit Comput. (2019) 33:1043–54. doi: 10.1007/s10877-019-00257-4, PMID: 30656507

[19] Liu C Wang LC Chang JF Lin KH Yeh YC Hsu PK . The role of extensive lymph node dissection in the new grading system for lung adenocarcinoma. Eur J Surg Oncol. (2024) 50:108540. doi: 10.1016/j.ejso.2024.108540, PMID: 39178686

[20] Bevilacqua Filho CT Schmidt AP Felix EA Bianchi F Guerra FM Andrade CF . Risk factors for postoperative pulmonary complications and prolonged hospital stay in pulmonary resection patients: a retrospective study. Braz J Anesthesiol. (2021) 71:333–8. doi: 10.1016/j.bjane.2021.02.003, PMID: 34229858 PMC9373437

[21] Wei W Zheng X Zhou CW Zhang A Zhou M Yao H . Protocol for the derivation and external validation of a 30-day postoperative pulmonary complications (PPCs) risk prediction model for elderly patients undergoing thoracic surgery: a cohort study in southern China. BMJ Open. (2023) 13:e066815. doi: 10.1136/bmjopen-2022-066815, PMID: 36764716 PMC9923300

[22] Tong C Shen Y Zhu H Zheng J Xu Y Wu J . Continuous relationship of operative duration with risk of adverse perioperative outcomes and early discharge undergoing thoracoscopic lung cancer surgery. Cancers (Basel). (2023) 15(2):371. doi: 10.3390/cancers15020371, PMID: 36672321 PMC9856387

[23] Kawanishi R Kakuta N Sakai Y Hari Y Sasaki H Sekiguchi R . Desflurane improves lung collapse more than propofol during one-lung ventilation and reduces operation time in lobectomy by video-assisted thoracic surgery: a randomized controlled trial. BMC Anesthesiol. (2022) 22:125. doi: 10.1186/s12871-022-01669-7, PMID: 35488195 PMC9052625

[24] Sha Y Xu R Shao S Yang J Tang B Liang Q . Tailored single-lung ventilation approaches and postoperative pulmonary outcomes in thoracic surgery. J Thorac Dis. (2025) 17:5371–87. doi: 10.21037/jtd-2025-314, PMID: 40809239 PMC12340303

[25] Yoshida N Watanabe M Baba Y Iwagami S Ishimoto T Iwatsuki M . Risk factors for pulmonary complications after esophagectomy for esophageal cancer. Surg Today. (2014) 44:526–32. doi: 10.1007/s00595-013-0577-6, PMID: 23584275

[26] Li X Yu L Yang J Fu M Tan H . Comparison of early postoperative pulmonary complications between two-lung ventilation with artificial pneumothorax and one-lung ventilation with bronchial blockade in patients undergoing minimally invasive esophagectomy: a retrospective propensity score-matched cohort study. J Thorac Dis. (2024) 16:1777–86. doi: 10.21037/jtd-23-1667, PMID: 38617773 PMC11009580

[27] Srinivas B Alluri K Fortuno P Rizzi M Suhail H Rhaleb N . Endothelial CHOP as a central mechanism in renovascular hypertension-induced vascular endothelial dysfunction and cardiac fibrosis. Cell Mol Life Sci. (2025) 82:232. doi: 10.1007/s00018-025-05741-6, PMID: 40512182 PMC12165939

[28] Kan HW Hsieh JH Chien HF Lin YH Yeh TY Chao CC . CD40-mediated HIF-1α expression underlying microangiopathy in diabetic nerve pathology. Dis Model Mech. (2018) 11(4):dmm033647. doi: 10.1242/dmm.033647, PMID: 29549140 PMC5963861

[29] Agar A Key S Yavuz H . Systemic inflammatory response index as a predictor of postoperative infectious complications in elderly patients undergoing posterior spinal instrumentation. J Clin Med. (2025) 14(21):7632. doi: 10.3390/jcm14217632, PMID: 41227027 PMC12608896

[30] Gómez-Ramirez S Jericó C Muñoz M . Perioperative anemia: Prevalence, consequences and pathophysiology. Transfus Apher Sci. (2019) 58:369–74. doi: 10.1016/j.transci.2019.06.011, PMID: 31416710

[31] Tadyanemhandu C Mukombachoto R Nhunzvi C Kaseke F Chikwasha V Chengetanai S . The prevalence of pulmonary complications after thoracic and abdominal surgery and associated risk factors in patients admitted at a government hospital in Harare, Zimbabwe-a retrospective study. Perioper Med (Lond). (2017) 6:11. doi: 10.1186/s13741-017-0066-3, PMID: 28852474 PMC5567626

[32] Homma T Saji H Shimada Y Tanabe K Kojima K Marushima H . Early chest tube removal within 6 hours after thoracic surgery results in improved postoperative prognosis and no adverse effects. J Thorac Dis. (2024) 16:3096–106. doi: 10.21037/jtd-23-1905, PMID: 38883671 PMC11170371

[33] Xu G Du J Zhang J Chen H Zheng B Yang Z . A propensity sore-matched study: Applying a modified chest tube drainage strategy in rapid rehabilitation following uni-portal thoracoscopic pulmonary wedge resection. Thorac Can. (2022) 13:1657–63. doi: 10.1111/1759-7714.14438, PMID: 35481947 PMC9161322

[34] Seder CW Salati M Kozower BD Wright CD Falcoz PE Brunelli A . Variation in pulmonary resection practices between the society of thoracic surgeons and the european society of thoracic surgeons general thoracic surgery databases. Ann Thorac Surg. (2016) 101:2077–84. doi: 10.1016/j.athoracsur.2015.12.073, PMID: 27021033

[35] Jammer I Wickboldt N Sander M Smith A Schultz MJ Pelosi P . Standards for definitions and use of outcome measures for clinical effectiveness research in perioperative medicine: European Perioperative Clinical Outcome (EPCO) definitions: a statement from the ESA-ESICM joint taskforce on perioperative outcome measures. Eur J Anaesthesiol. (2015) 32:88–105. doi: 10.1097/EJA.0000000000000118, PMID: 25058504

