# Supplementary material for: A machine learning-based predictive model for postoperative pulmonary complications in lung cancer and its SHAP interpretation
Source: Front Oncol. 2026 Mar 13;16:1749808. doi: 10.3389/fonc.2026.1749808 (PMC13022757; doi:10.3389/fonc.2026.1749808)
Supplement: Supplementary file 1 [file DataSheet1.docx]

Supplementary Material

To further enhance reproducibility and transparency, the Supplementary Materials provide an expanded and structured presentation of the key steps and supporting evidence for data preprocessing, cohort characterization, and model interpretation beyond what is reported in the main text. Specifically, Supplementary Figure S1 compares the distributions of continuous variables before and after missing-value imputation, showing that the central tendency and dispersion remain broadly consistent after non-parametric iterative imputation using MissForest, supporting the main-text statement that no systematic distortion was introduced by imputation . Supplementary Figure S2 visualizes the distributions of major continuous predictors stratified by PPCs (POC: Yes/No), providing an intuitive depiction of group differences consistent with the direction of the primary analyses, while Supplementary Figure S3 summarizes the frequency distributions of core categorical variables to contextualize sample composition .

In addition, the supplement extends model-level evidence: Supplementary Figure S4 reports the decision curve analysis and calibration curve of the full-feature (20-variable) KNN model, allowing direct comparison with the parsimonious model selected in the main manuscript; Supplementary Figure S5 presents a SHAP dependence plot for SIRI colored by operation time, offering an interpretable view of how inflammatory burden may relate to predicted PPC risk across different surgical durations . Complementary to these figures, three supplementary tables are provided. Supplementary Table S1 details the clinical definitions, data sources, units/ranges, coding/grouping rules, perioperative timing (pre-/intraoperative), and formulas for all candidate predictors (including BMI and the SIRI formula), ensuring consistent variable operationalization. Supplementary Table S2 compares predictive performance between the 11-variable KNN model and the full 20-variable KNN model in both training and independent test sets (AUC with 95% CI, accuracy, NPV, PPV, sensitivity, specificity), highlighting that adding variables produced perfect apparent training performance but did not improve test-set discrimination, consistent with overfitting concerns. Supplementary Table S3 summarizes the missingness profile across all 20 variables, demonstrating near-complete data for categorical variables and minimal missingness for most continuous measures, with missing values mainly concentrated in pulmonary-function parameters (FEV1% and DLCO%), thereby supporting the manuscript’s missing-data handling strategy . All analyses and figure generation were conducted in R 4.4.2, with continuous variables Z-score standardized before modeling and missing values imputed using MissForest, while full details of variable selection and preprocessing are described in the Methods section of the main text .

1. **Supplementary Figures**

**1.1 Supplementary Figure 1:Comparison of the distributions of continuous variables before and after missing value imputation.**

The figure compares the distribution patterns of seven consecutive indicators before and after imputation, including: white blood cell count (WBC), systemic inflammatory response index (SIRI), platelet count (PLT), hemoglobin (HB), albumin (ALB), percentage of forced expiratory volume in one second (FEV1%), and percentage of diffusion capacity (DLCO%). The left column shows the distribution of the original data, while the right column displays the distribution after imputation using MissForest. Overall, the central tendency and dispersion of each variable after imputation remain consistent with the original data, with no observed systematic drift or abnormal peak/trough, suggesting that MissForest effectively preserved the true distribution characteristics in this dataset, which aligns with our description in the main text that there are "no significant differences in data before and after imputation." This result supports the use of the complete dataset after imputation in subsequent feature selection and modeling stages.


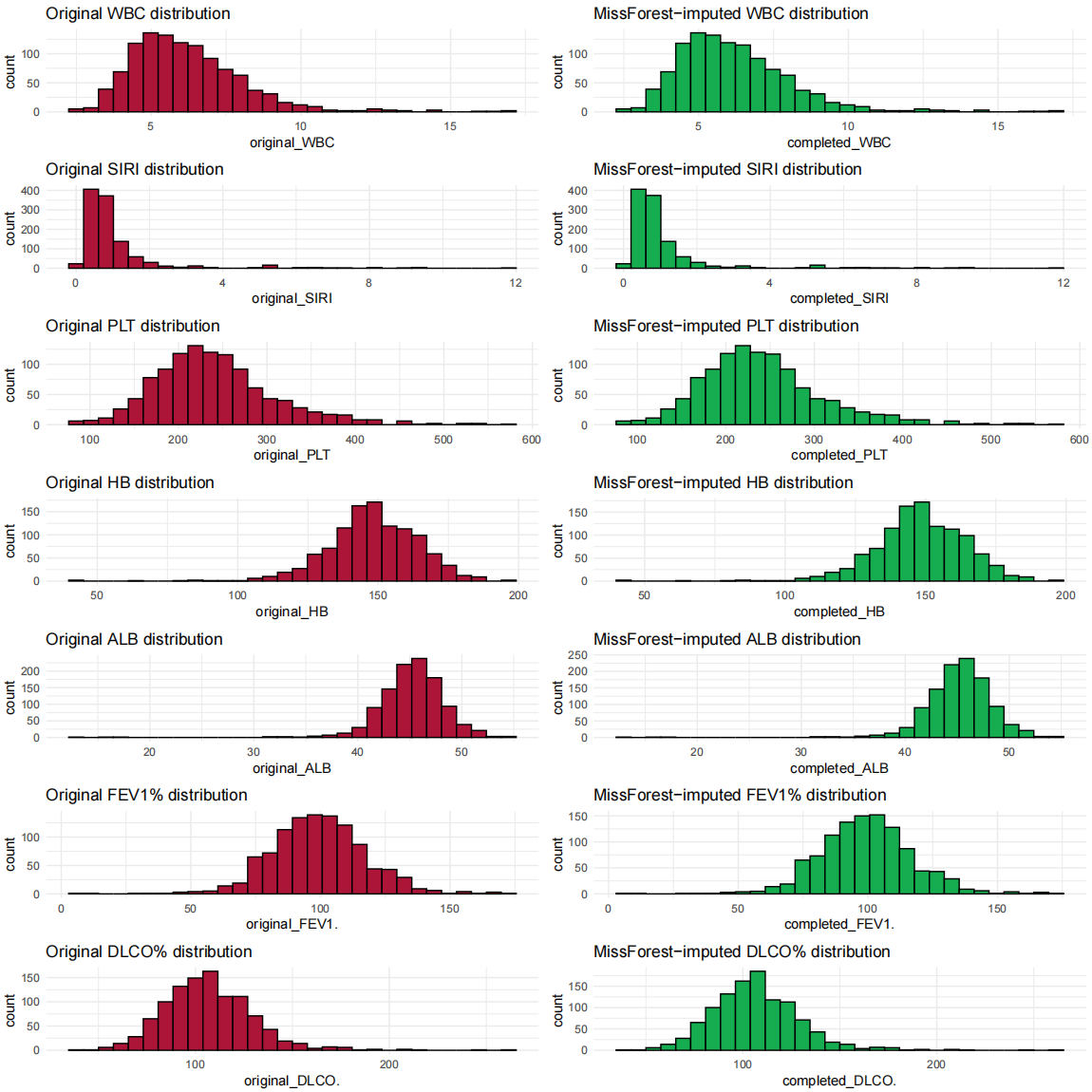


Supplementary Figure 1 Comparison before and after data imputation

**1.2 Supplementary Figure 2: Distributions of continuous variables stratified by the occurrence of PPCs.**

To intuitively illustrate the potential differences related to complications, we plotted box plots of 11 continuous variables based on the occurrence of PPCs (marked in the figure as "POC: Yes/No"): age, BMI, surgical time, anesthesia time, WBC, SIRI, PLT, HB, ALB, FEV1%, and DLCO%. From the distribution trends, it can be observed that the complication group had an overall older age and longer surgical and anesthesia times; inflammatory-related indicators (such as SIRI) exhibited higher median values and a wider range of dispersion in the complication group; nutritional and hematological indicators (such as HB and ALB) were slightly lower in the complication group. These visual conclusions are consistent with the direction of the single/multivariate analyses in the main text, providing a vivid support for the primary results.


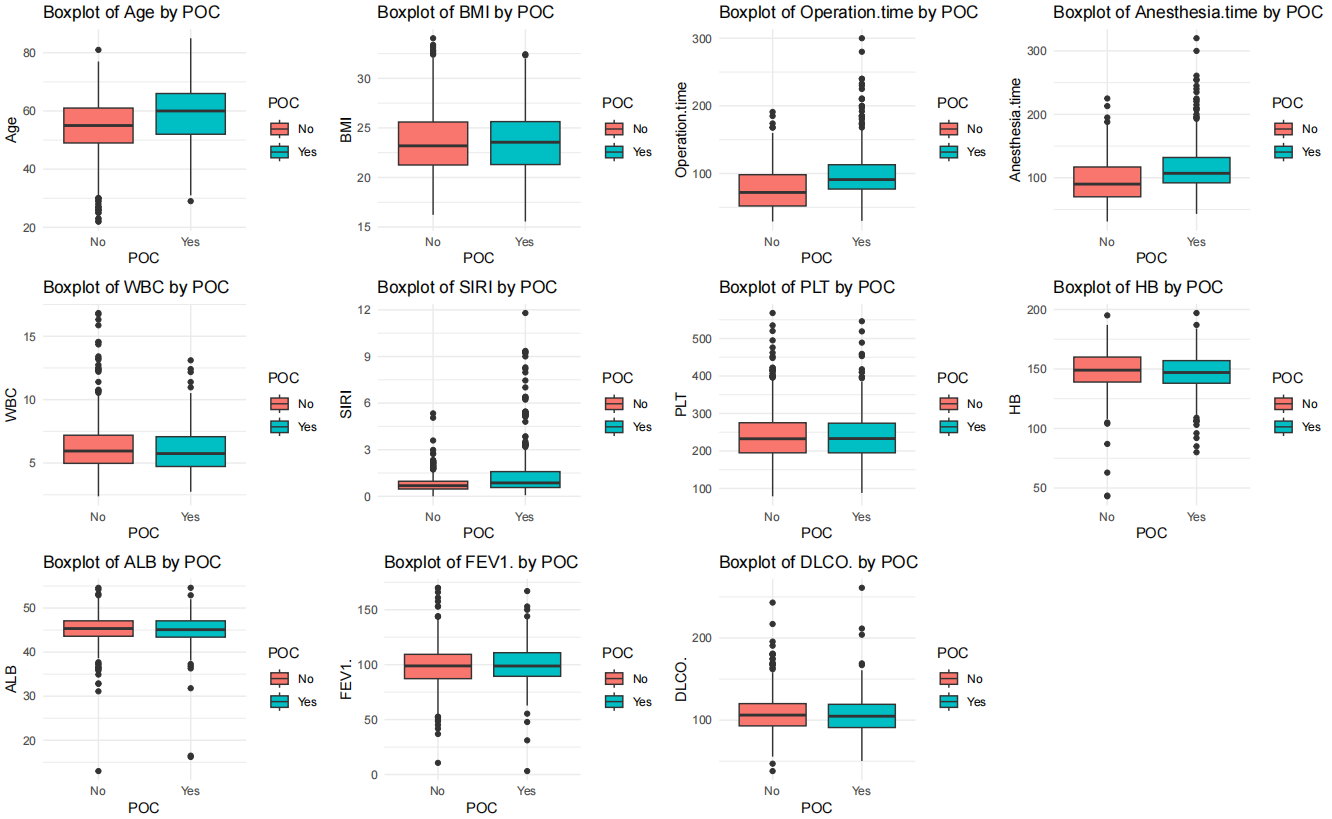


Supplementary Figure 2: Descriptive statistics of count data

**1.3 Supplementary Figure 3: Frequency distribution of major categorical variables.**

The figure presents a count bar chart for nine categorical variables: Gender, Past Surgical History (PSH), Hypertension, Diabetes, Coronary Heart Disease (CHD), Smoking, Surgical Site, whether Lymph Node Dissection (LND) was performed, and ASA classification. The overall distribution characteristics indicate that the sample predominantly consists of females and individuals classified as ASA II. Most patients do not have a PSH, CHD, Diabetes, or Hypertension, although a certain proportion are smokers. There is a slight predominance of surgeries performed on the right side. The counts for LND are nearly equal between 'Yes' and 'No'. This frequency overview serves to illustrate the composition of the sample and potential co-linear distributions, providing context for subsequent model training and external comparability assessments.


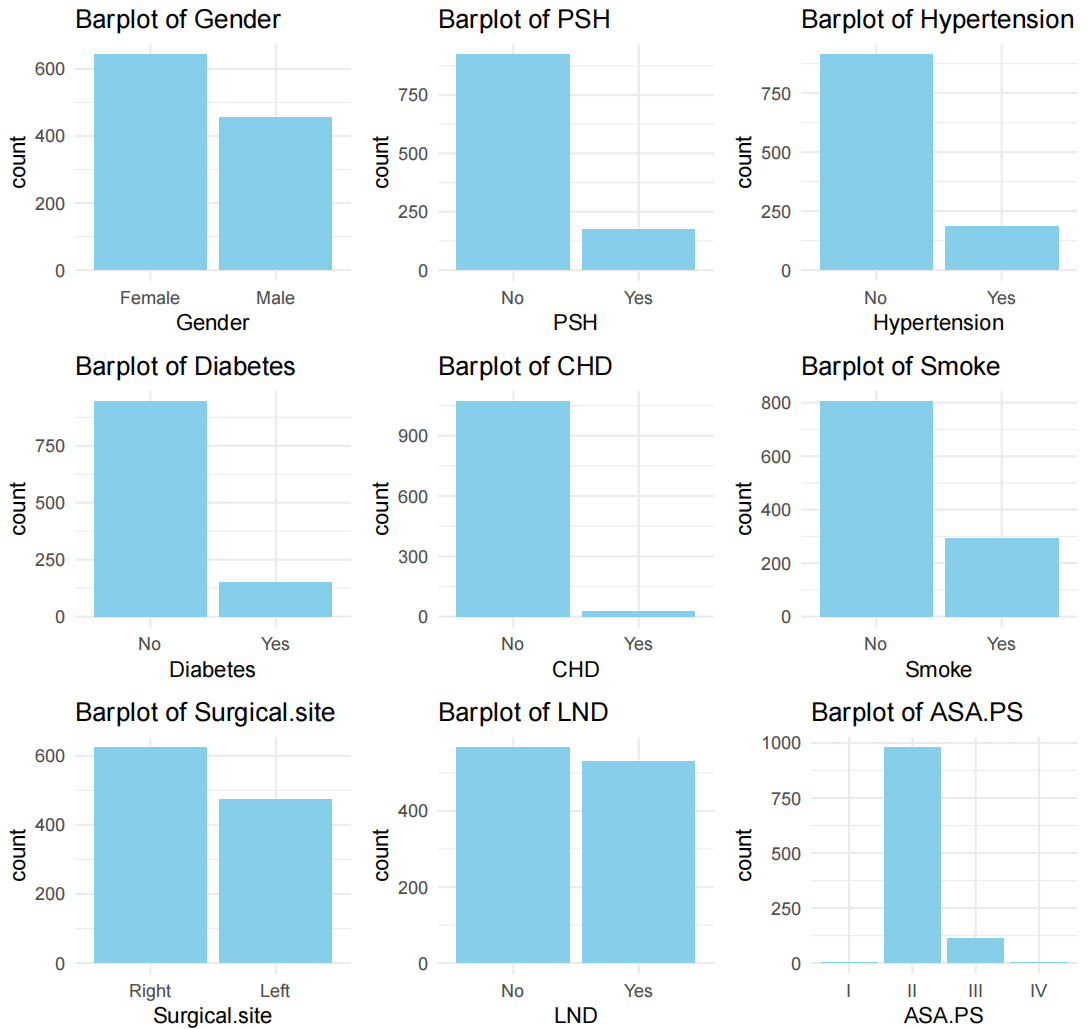


Supplementary Figure 3: Descriptive statistics of categorical data

**1.4 Supplementary Figure 4: DCA and calibration curves for the full-feature model of all variables.(A) DCA. (B) Calibration curve.**

Supplementary Figure S4 (All-variable KNN model: decision-curve analysis and calibration). Supplementary Figure S4 presents the clinical utility and calibration performance of the full 20-variable KNN model under the same preprocessing, hyperparameter-tuning strategy, and train/test split used in the main analyses, with model development conducted via 10-fold cross-validation in the training set and a one-time independent evaluation in the test set . Panel A shows the decision curve analysis (DCA) for the all-variable KNN model; consistent with the main-text KNN results, the model provides net benefit over “treat-all” and “treat-none” strategies within clinically relevant threshold ranges, supporting its potential value for perioperative risk stratification . Panel B displays the calibration curve, illustrating the agreement between predicted probabilities and observed event rates; overall, the calibration pattern of the full-feature model is similar to that of the 11-variable KNN model reported in the main text . Importantly, although the full-feature model fit the training data extremely well, it did not improve generalization in the test set (AUC decreased from 0.807 to 0.787, with sensitivity increasing but specificity decreasing), and its calibration and DCA curves remained broadly comparable to the 11-variable model . Therefore, considering generalization risk, model complexity, and the clinical burden of collecting additional variables, the 11-variable KNN model was retained as the primary model in the main text, while the full-feature results are provided here as supplementary evidence .


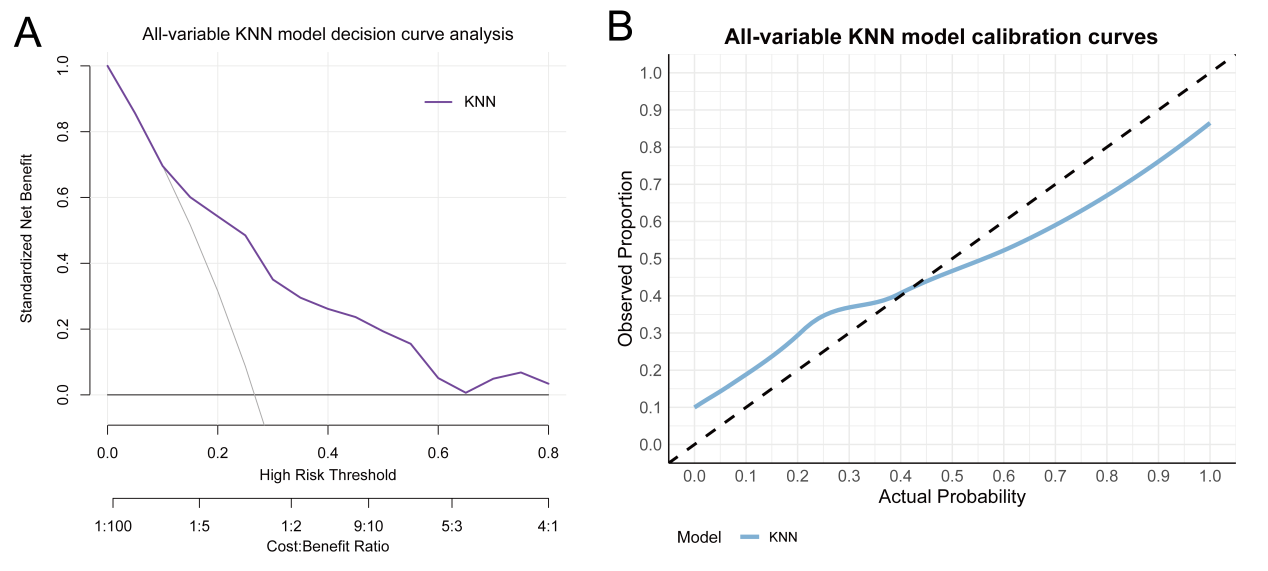


Supplementary Figure 4: DCA and calibration curves for the full-feature model of all variables.(A) DCA. (B) Calibration curve

**1.5 Supplementary Figure 5: SHAP dependence plot of SIRI in the KNN model (colored by operative duration).**

Supplementary Figure S5 shows a SHAP dependence scatter plot for the KNN model, with SIRI on the x-axis and its corresponding SHAP value on the y-axis; points are color-coded by operation time to visualize how the effect of SIRI may vary across different levels of surgical exposure. Overall, when SIRI is low (approximately <2), SHAP values cluster around zero with little apparent variation by operation time. In contrast, when SIRI increases (approximately >3), SHAP values rise markedly, indicating a stronger contribution of higher inflammatory burden to the predicted risk of PPCs. Moreover, at comparable SIRI levels, longer operation time tends to coincide with higher SHAP values, suggesting a possible synergistic pattern whereby “high inflammation + prolonged surgical exposure” amplifies PPC risk. This pattern is consistent with the main-text discussion that SIRI is an important predictor and may act jointly with perioperative exposure, although SHAP primarily provides model-based association and interaction signals; therefore, the observed interaction should be further confirmed using explicit interaction-term modeling or stratified analyses.


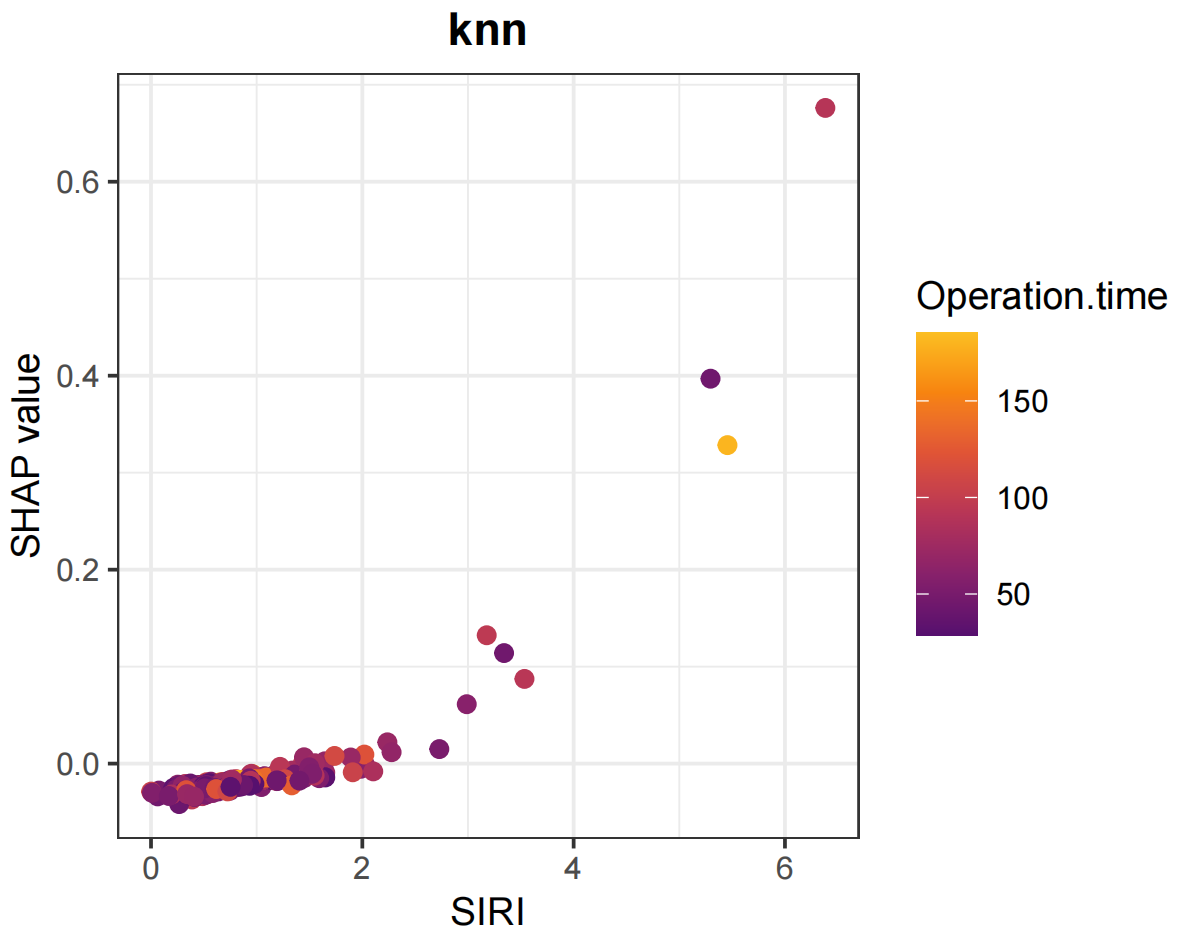


Supplementary Figure 5: SHAP dependence plot of SIRI in the KNN model (colored by operative duration).

1. **Supplementary Tables**
   1. **Supplementary Table 1: Variable Description Table**

Supplementary Table S1 summarizes the 20 prespecified candidate predictors used in the main analyses and provides their clinical definitions, data sources, units/ranges, coding/grouping rules, perioperative time window (pre-/intraoperative), and key remarks/formulas. The candidate set spans (i) baseline demographics and comorbidity history (sex, age, past surgical history, hypertension, diabetes, CHD, and smoking), (ii) perioperative/anesthetic characteristics (surgical side, lymph node dissection, ASA physical status, operation time, and anesthesia time), and (iii) objective preoperative laboratory and pulmonary-function indicators (WBC, PLT, hemoglobin, albumin, FEV1%, DLCO%) as well as an inflammation-derived composite marker (SIRI, calculated as neutrophil × monocyte / lymphocyte). Categorical variables were uniformly binarized (e.g., No=0/Yes=1; Female=0/Male=1; Right=0/Left=1), ASA-PS was treated as an ordered variable (I–IV coded 1–4), and BMI was recorded continuously but also grouped according to Chinese BMI criteria (<18.5, 18.5–23.9, 24.0–27.9, ≥28 kg/m²) to align with the stratification strategy described in the main text. To ensure clinical validity and temporal consistency, laboratory and pulmonary-function values were taken from the most recent preoperative assessments (e.g., CBC within 7 days before surgery), operation time was abstracted preferentially from surgical records, and key history variables (e.g., prior surgery) were verified against both self-report and medical documentation. This table therefore operationalizes the variable definitions used throughout model development and interpretation in the main manuscript, enabling reproducibility and transparent clinical mapping of each predictor.

| **Variable (in manuscript)** | **Clinical definition & source** | **Unit & range** | **Coding & grouping** | **Pre/Intra/Post** | **Remarks/Formula/References** |
| --- | --- | --- | --- | --- | --- |
| Gender | Biological sex; Source: Medical record cover page/Admission registration information | Categorical variable; Female/Male | Female=0，Male=1 | Preoperative | — |
| PSH | Past surgical history; Source: Admission medical history/Preoperative assessment form | Categorical variable; No/Yes | No=0，Yes=1； | Preoperative | Verified by comparing patient self-reports with medical records |
| Hypertension | History of hypertension diagnosis; Source: Medical history + Medication history + Past diagnoses | Categorical variable; No/Yes | No=0，Yes=1； | Preoperative | — |
| Diabetes | History of diabetes diagnosis; Source: Medical history + Medication history + Past diagnoses | Categorical variable; No/Yes | No=0，Yes=1； | Preoperative | — |
| CHD | Coronary heart disease; Source: Medical history/Discharge diagnosis/Interventional or imaging records | Categorical variable; No/Yes | No=0，Yes=1； | Preoperative | — |
| Smoke | Smoking history; Source: Admission medical history/Anesthesia assessment | Categorical variable; No/Yes | No=0，Yes=1； | Preoperative | Current or former smoking counts as Yes. |
| Surgical site | Surgical side; Source: Surgical record/Anesthesia record | Categorical variable; Right/Left | Right=0，Left=1 | Intraoperative | — |
| LND | Lymph node dissection; Source: Surgical record + Pathology report | Categorical variable; No/Yes | No=0，Yes=1 | Intraoperative | Systematic cleaning counts as Yes |
| ASA PS | ASA Physical Status classification; Source: Anesthesia preoperative assessment form | Ordered categories; I–IV | I=1，II=2，III=3，IV=4 | Preoperative |  |
| Age | Age; Source: ID card/Medical record cover page | years; based on actual NAP standards | Continuous variable | Preoperative | Calculated as of the day of surgery |
| **Variable (in manuscript)** | **Clinical definition & source** | **Unit & range** | **Coding & grouping** | **Pre/Intra/Post** | **Remarks/Formula/References** |
| BMI | Body Mass Index (BMI); Source: Preoperative height and weight measurement | kg/m²; Common range: 10–60 | Continuous variable; grouped as follows: <18.5, 18.5–23.9, 24.0–27.9, ≥28 (per Chinese standards) | Preoperative | BMI = Weight (kg) / Height (m) |
| Operation time | Surgery duration; Source: Anesthesia/Surgical record | Minutes (min) | Continuous variable | Intraoperative | The surgical record shall prevail. |
| Anesthesia time | Anesthesia duration; Source: Anesthesia record | Minutes (min) | Continuous variable | Intraoperative | From the start of anesthesia to the end of anesthesia |
| WBC | White blood cell count; Source: Final preoperative complete blood count (CBC) | ×10⁹/L; Common range: 0–50 | Continuous variable | Preoperative | Most recent preoperative complete blood count (within 7 days prior to surgery) |
| PLT | Platelet count; Source: Final preoperative complete blood count (CBC) | ×10⁹/L; Common range: 0–1000 | Continuous variable | Preoperative | — |
| HB | Hemoglobin; Source: Final preoperative complete blood count (CBC) | g/L; Common range: 30–200 g/L | Continuous variable | Preoperative | — |
| ALB | Serum Albumin; Source: Final Preoperative Biochemical Test | g/L; Common range: 10–60 g/L | Continuous variable | Preoperative | — |
| FEV1% | Forced Expiratory Volume in One Second as a Percentage of Predicted Value; Source: Pulmonary Function Test Report | %; Common range: 10–150 | Continuous variables | Preoperative | The most recent preoperative pulmonary function test |
| DLCO% | Carbon Monoxide Diffusion Capacity as a Percentage of Predicted Value; Source: Pulmonary Function Test Report | %; Common range: 10–150 | Continuous variable | Preoperative | — |
| SIRI | Systemic Inflammation Response Index; Source: Differential Blood Count (Absolute Values of Neutrophils/Monocytes/Lymphocytes) | Typically used as a dimensionless indicator | Continuous variables | Preoperative | SIRI = Neutrophil count × Monocyte count / Lymphocyte count |

**2.2 Supplementary Table 2: Performance comparison between the 11-variable KNN model and the full (20-variable) KNN model.**

Supplementary Table S2 summarizes the key discrimination and classification metrics reported in the main manuscript for two feature-set strategies—an 11-variable model and a full 20-variable model—evaluated in both the training set and an independent test set. Metrics include AUC with 95% confidence intervals, accuracy, NPV, PPV, sensitivity, and specificity, allowing assessment of whether adding more variables yields stable gains in generalization performance. Overall, the 11-variable model achieved strong apparent performance in the training set (AUC 0.960, 95% CI 0.948–0.972; accuracy 0.889). In the test set, discrimination decreased to an AUC of 0.807 (95% CI 0.775–0.859), with a pattern of high specificity (0.942) but low sensitivity (0.33), indicating a classification tendency toward minimizing false positives and providing relatively strong rule-out capability (NPV 0.794). In contrast, the full-variable model showed perfect fit in the training set (AUC 1.000 with all metrics equal to 1.00), but its test-set AUC was only 0.787 (95% CI 0.732–0.842), offering no improvement over the 11-variable model and suggesting substantial overfitting and limited generalizability. Notably, in the test set, sensitivity increased from 0.33 to 0.42 when using all variables, but specificity decreased from 0.942 to 0.917, reflecting the expected trade-off of slightly improved case detection at the cost of more false positives. Taken together with the main-text considerations of robustness, clinical feasibility, and data-collection burden, Supplementary Table S2 further supports that the parsimonious 11-variable strategy provides comparable or better generalization performance, whereas simply increasing the number of predictors does not necessarily enhance test-set discrimination.

| **ML model** | **AUC(95%CI)** | **Accuracy** | **NPV** | **PPV** | **Sensitivity** | **Specificity** |
| --- | --- | --- | --- | --- | --- | --- |
| **11 variables** |  |  |  |  |  |  |
| Train | 0.960(0.948-0.972) | 0.889 | 0.883 | 0.922 | 0.611 | 0.983 |
| Test | 0.807(0.775-0.859) | 0.778 | 0.794 | 0.674 | 0.33 | 0.942 |
| **all variables** |  |  |  |  |  |  |
| Train | 1.000(1.000-1.000) | 1 | 1 | 1 | 1 | 1 |
| Test | 0.787(0.732-0.842) | 0.784 | 0.813 | 0.649 | 0.42 | 0.917 |

**2.3 Supplementary Table 3: Summary table of missingness for 20 variables.**

Supplementary Table S3 reports the extent of missingness for each candidate predictor used in the main analyses, stratified by categorical and continuous variables. Overall, missingness was minimal, and all categorical variables(POC, gender, PSH, hypertension, diabetes, CHD, smoking history, surgical site, LND, and ASA-PS) had 0 missing values (0%), indicating complete recording of baseline history and perioperative categorical information. Among continuous variables, age, BMI, operation time, and anesthesia time were complete (0% missing), while routine laboratory indices (WBC, PLT, HB, ALB) and SIRI each had only 1 missing observation (0.09%), suggesting negligible impact on model training and evaluation. The only variables with non-trivial missingness were pulmonary-function parameters, with FEV1% missing in 38 cases (3.46%) and DLCO% missing in 44 cases (4.01%), consistent with the main-text statement that lung-function testing may be unavailable in a small subset of patients due to clinical feasibility or incomplete preoperative assessment. In line with the data-handling strategy described in the manuscript, this overall low level of missingness supports the robustness of the modeling pipeline and reduces the likelihood that imputation (if applied) would materially bias performance estimates, while highlighting pulmonary-function measures as the primary source of missing data.

| Categorical variables | | | Continuous variables | | |
| --- | --- | --- | --- | --- | --- |
| Variable | Missing (n) | Missing (%) | Variable | Missing (n) | Missing (%) |
| POC | 0 | 0% | Age | 0 | 0.00% |
| Gender | 0 | 0% | BMI | 0 | 0.00% |
| PSH | 0 | 0% | Operation time | 0 | 0.00% |
| Hypertension | 0 | 0% | Anesthesia time | 0 | 0.00% |
| Diabetes | 0 | 0% | WBC | 1 | 0.09% |
| CHD | 0 | 0% | SIRI | 1 | 0.09% |
| Smoke | 0 | 0% | PLT | 1 | 0.09% |
| Surgical site | 0 | 0% | HB | 1 | 0.09% |
| LND | 0 | 0% | ALB | 1 | 0.09% |
| ASA-PS | 0 | 0% | FEV1% | 38 | 3.46% |
|  |  |  | DLCO% | 44 | 4.01% |
